# Unveiling the Structure of PROT and ATB^0,+^: Unique Members of the Glycine Transporter Subfamily

**DOI:** 10.3390/molecules30224412

**Published:** 2025-11-14

**Authors:** Dorota Stary, Marek Bajda

**Affiliations:** 1 Department of Physicochemical Drug Analysis, Faculty of Pharmacy, Jagiellonian University Medical College, Medyczna 9, 30-688 Cracow, Poland; dorota.stary@doctoral.uj.edu.pl; 2 Doctoral School of Medical and Health Sciences, Jagiellonian University Medical College, Św. Łazarza 16, 31-530 Cracow, Poland; 3 Institute of Pharmacy and Food Chemistry, University of Würzburg, Am Hubland, 97074 Würzburg, Germany

**Keywords:** SLC6A7, SLC6A14, glycine transporter subfamily, structure, inhibitors, homology modelling, docking, molecular dynamics

## Abstract

The proline transporter (PROT, SLC6A7) and the neutral and cationic amino acid transporter (ATB^0,+^, SLC6A14) belong to the glycine transporter subfamily, exhibiting distinct substrate specificities and physiological functions. PROT modulates neurotransmission through proline transport in the brain, while ATB^0,+^ facilitates nutrient uptake, especially in the gastrointestinal tract. Impaired function of PROT has been associated with neurological disorders, while ATB^0,+^ overexpression has been linked to cancers. Despite their biological relevance, the pool of known ligands for these transporters is limited, and their exact 3D structures remain unknown. Therefore, we conducted an in silico analysis of PROT and ATB^0,+^ and compared the obtained results with available literature data on the glycine transporter GlyT1, from the same subfamily. Using homology modelling, docking studies, and molecular dynamics simulations, we investigated the structural properties of PROT and ATB^0,+^ and described protein–ligand interactions. We pointed crucial residues responsible for ligand binding, including Tyr133, Tyr297, Phe303, and Phe403 in PROT and Trp327, Val128, and Tyr321 in ATB^0,+^. This work provides new insights into the molecular features of PROT and ATB^0,+^ transporters, which could support the development of novel transporter inhibitors.

## 1. Introduction

The solute carrier 6 (SLC6) family uses a Na^+^ ion gradient to actively transport substrates across cell membranes [1]. This family consists of 20 transporters (SLC6A1—SLC6A20) which are divided into four groups: GABA, monoamine, glycine (neurotransmitter), and neutral (nutrient) amino acid transporters [1]. Among the proteins belonging to the glycine transporter subfamily, the proline transporter (PROT, SLC6A7) and the neutral and cationic amino acid transporter (ATB^0,+^, SLC6A14) are less explored but seem to be promising targets for novel drugs [2].

PROT translocates proline from the extracellular space into the neuronal cytoplasm. It is suggested that SLC6A7 is involved in the modulation of glutamatergic transmission, since L-proline contributes to the activity of NMDA (N-methyl-D-aspartic acid), AMPA (α-amino-3-hydroxy-5-methyl-4-isoxazolepropionic acid), and kainic acid receptors [3]. PROT is present in several tissues, but its highest expression is observed in the brain, where it is known as a “brain-specific proline transporter” [4].

ATB^0,+^ transports neutral and cationic amino acids, showing the highest preference for those with nonpolar side chains and lower affinity toward polar or charged ones. Substrates, ranked by increasing EC_50_, include hydrophobic amino acids (Ile, Leu, Met, Phe, Trp, Val), followed by more polar ones (Ser, Tyr), small, nonpolar (Ala), and basic residues (His, Lys, Arg). The lowest affinities are observed for: Gly, Cys, Asn, Thr, and Gln. ATB^0,+^ also shows some affinity for proline, but it has been considered biologically irrelevant [5,6]. The primary physiological role of ATB^0,+^ is the absorption of amino acids from the gastrointestinal tract [5]. Its expression has been detected in the salivary glands, stomach, colon, appendix, and at lower levels in the duodenum. It is also observed in the respiratory system with the highest level in the lungs, followed by the trachea [5,7,8].

As PROT is considered a “brain-specific” transporter, it is implicated in neurological disorders, although its role remains under investigation. Through the ability of L-proline to activate and modulate NMDA and AMPA receptors, PROT is thought to play an indirect but important role in regulating glutamatergic neurotransmission. Disruption of this pathway has been linked to schizophrenia and to neuronal damage following ischemic stroke [9,10]. Consistent with the glutamatergic hypothesis of schizophrenia, which associates NMDA receptor hypofunction with negative psychotic symptoms, PROT may influence disease pathology by regulating proline availability and thus NMDA receptor activity. In a ketamine-induced psychosis model, inhibition of PROT with the compound LQFM215 effectively reduced hyperlocomotion, enhanced social interaction, and prevented sensorimotor gating deficits, supporting its therapeutic potential in schizophrenia model [10]. In ischemic stroke, NMDAR-mediated excitotoxicity is a major cause of neuronal death. After stroke, plasma L-proline levels are reduced, while higher circulating levels correlate with better functional recovery. In a mouse model, the PROT inhibitor LQFM215 lowered hippocampal proline uptake and reduced infarct size. It also improved motor outcomes without affecting anxiety-like or memory-related behaviours, indicating a neuroprotective effect [9]. Beyond these findings, PROT overexpression has been detected in Friedreich’s ataxia, a neurodegenerative disease characterized by muscle weakness, impaired coordination, sensory loss, and cardiomyopathy, although further studies are needed to clarify its role [11]. In addition to changes in transporter expression, rare inherited variants of *SLC6A7* provide further evidence of its importance for normal brain development and function. Very rare, homozygous loss-of-function variants in *SLC6A7*, together with *MPPE1* (Metallophosphoesterase 1) mutations, have been identified in a complex neurodevelopmental disorder characterized by severe developmental delay, generalized dystonia with episodic status dystonicus, chorea, epilepsy, and cataracts [12]. Functional studies demonstrated that the PROT variant reduces cell-surface expression and proline transport, pointing to the role of PROT in normal motor and neurodevelopmental function [12]. Moreover, alterations in the SLC6A7 gene have been proposed as a potential biomarker for autism diagnosis [13], although evidence is still limited. In summary, PROT inhibition may represent a promising research direction in neurological disorders. 

While PROT is considered a target in neurological disorders, ATB^0,+^, due to its overexpression, is implicated in various cancers, including estrogen receptor-positive breast (ERBC), cervical (CR), colorectal (CRC), and pancreatic (PC) cancers [14,15,16,17,18]. High protein levels have been associated with advanced neoplasm stage, metastasis, and poor prognosis in patients. In several studies, the therapeutic potential of ATB^0,+^ inhibitor—α-methyl-tryptophan (α-MT) was tested. The compound has shown antitumour activity in cancer cell lines and mouse models [14,16,17,18,19]. In estrogen receptor-positive breast cancer, *SLC6A14* expression was approximately nine-fold higher than in normal tissue and supported tumour growth by supplying leucine and arginine [16]. Leucine is a key activator of mTOR kinase, a known target in anticancer therapy. Inhibition of SLC6A14 using α-MT in ERBC cells reduced nutrient availability and blocked mTOR kinase activity, which led to cell starvation, activation of autophagy, and then caused cancer cell apoptosis [16]. In cervical cancer, *SLC6A14* expression was upregulated up to 5.6-fold accompanied by elevated inducible nitric oxide synthase (iNOS) levels, suggesting that enhanced arginine transport by ATB^0,+^ may contribute to increased nitric oxide production in the tumour microenvironment [17]. Strong association between *SLC6A14* upregulation and tumour progression has been reported in colorectal cancer [15]. Transporter due to increased amino acid supply, activates the Akt–mTOR signalling pathway to promote tumour progression, while its blockade with inhibitor suppresses CRC growth [19]. Tumorigenic effects were also likely mediated by activation of the JAK2/JAK3 kinase signalling pathway, and blocking the SLC6A14/JAK2/JAK3 axis has therefore been proposed as a potential therapeutic strategy for CRC [14]. In pancreatic cancer, protein expression rises dramatically (13- to 163-fold), and in this case SLC6A14 has been identified as a novel PC biomarker [20]. Collectively, these studies demonstrate that transporter ATB^0,+^ may represent a potential target for future anticancer research, particularly in breast, colorectal, and pancreatic cancers.

Upregulation of ATB^0,+^ has also been linked to non-cancerous diseases. Its overexpression has been observed in Crohn’s disease, and the treatment with the inhibitor α-MT has shown promising effects. In a mouse model, α-MT downregulated SLC6A14, suppressed pro-inflammatory cytokines, and improved intestinal integrity, highlighting its therapeutic potential [21]. On the other hand, genetic variants caused by SNP in the *SLC6A14* gene have been associated with obesity, cystic fibrosis, and male infertility, although these links require further studies [7,22,23,24].

The overall structure of PROT and ATB^0,+^ transporters is consistent with those of other proteins in the glycine transporter subfamily and the broader SLC6 family. However, PROT and ATB^0,+^ exact 3D structures remain unknown. Knowledge about these structures could facilitate the development of new ligands. Therefore, we decided to thoroughly characterize the selected transporters. Here, we present the structure of PROT and ATB^0,+^ obtained through homology modelling, along with the binding modes of their substrates and inhibitors. Our study provides insight into the structural features of transporters and identifies key residues involved in ligand binding.

## 2. Results and Discussion

Homology modelling was employed to generate 3D structural models of the PROT and ATB^0,+^ transporters. We performed sequence alignment, model construction, and evaluation to identify the most accurate models. These were subsequently used for docking studies and molecular dynamics simulations and were also compared with other transporters from the SLC6 family, particularly GlyT1 [25].

### 2.1. Model Building and Evaluation

To select appropriate templates, we assessed the sequence identity and similarity between proteins from the glycine subfamily and SLC6 members with available structures in the Protein Data Bank (PDB) [26] (Table 1). The PROT transporter revealed the highest identity and similarity with the human GlyT1 transporter (*h*GlyT1), with values of 43.1% and 60.2%, respectively. ATB^0,+^ exhibited the highest identity with the *Drosophila melanogaster* DAT structure (*d*DAT)(40.1%) and human GAT1 (*h*GAT1) (38.5%) (Table 1). PROT and ATB^0,+^ shared approximately 30% identity with human SIT1 (*h*SIT1), similar to the values observed for human GlyT1 and GlyT2 (Table 1).

We observed that the bacterial homologue LeuT (leucine transporter) had the lowest amino acid sequence similarity with other transporters (~20%). Despite this, we included it in our homology modelling studies, particularly for ATB^0,+^, as both *Aquifex aeolicus* LeuT (*a*LeuT) and ATB^0,+^ transport leucine. For homology modelling, we selected 14 experimental structures representing various conformational states, including outward-open, outward-occluded, inward-occluded, and inward-open. As templates, we selected: structures of *a*LeuT, *d*DAT, *h*SERT, *h*GlyT, and *h*GAT1 (Table S1). The generated models were evaluated using DOPEscore, QMEAN, and Ramachandran plot. These tools evaluate multiple structural parameters that reflect the quality of the models and their consistency with experimentally resolved protein structures. For each transporter (PROT and ATB^0,+^), the eight highest-quality models representing four distinct conformational states, were selected (exemplary models are shown in Figures S1 and S2). Overall, the models showed beneficial DOPEscore and QMEAN values, comparable to those of the templates deposited in the PDB (Table S2). Among selected models for each transporter, the highest-ranked ones were built on the following templates: in outward-open state—4XP9 and 6M2R; outward-occluded—4XPH (*d*DAT); inward-occluded—7Y7W (*h*GAT1); and inward-open—6ZBV (*h*GlyT1) (Table S2). This result is consistent with the sequence analysis, as *d*DAT, *h*GlyT1, and *h*GAT1 share the greatest sequence identity with PROT and ATB^0,+^. The stereochemical quality of the models was confirmed, with over 90% of residues falling within the favoured regions in Ramachandran plots (Figures S3 and S4).

### 2.2. Structure of PROT and ATB^0,+^ Transporters

#### 2.2.1. Overall Structure and Mechanism of Action

**Figure 1:**
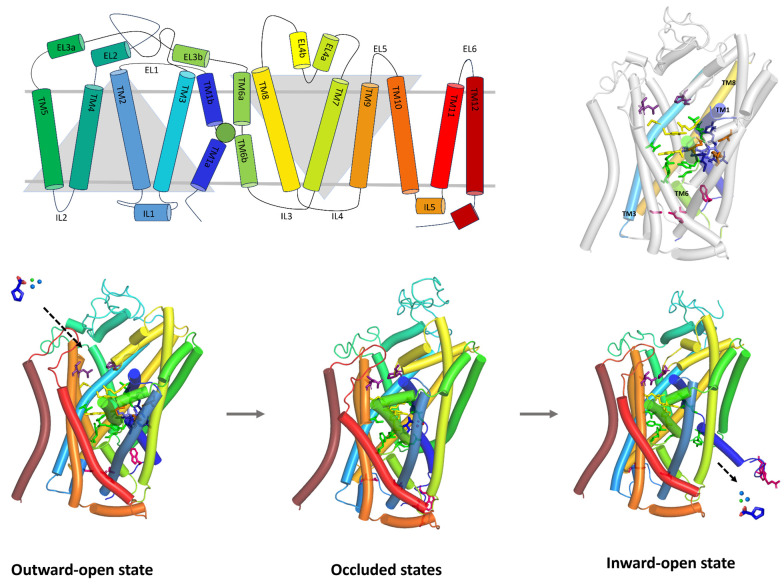
Topology of the glycine subfamily member—PROT (upper, left panel). The binding site is represented as a green sphere. Homology model of PROT in outward-open state (upper, right panel). Schematic representation of the transport mechanism (down panel). Proline—dark blue sticks, blue and green spheres—ions. Domains creating S1 binding site are coloured as follows: TM3—cyan, TM1—dark blue, TM6—split pea, TM8—yellow. Residues are marked with the colours: extracellular gate—yellow, intracellular gate—pink, Na^+^1 and Na^+^2 binding site—blue, Cl^−^ binding site—orange, S2 site binding site—violet, S1 binding site—green.

PROT and ATB^0,+^ share the general structural features characteristic of the SLC6 family. Similarly to GlyT1, which belongs to the same subfamily, they consist of 12 transmembrane helical domains (TMs) connected by extracellular (EL) and intracellular loops (IL). The core of these transporters is formed by two groups of helices, TM1-TM5 and TM6-TM10, arranged with a pseudo-two-fold axis of symmetry (Figure 1, right, upper panel). This structural organization is consistent across SLC6 family members [1]. It was observed that PROT and ATB^0,+^ models have a longer EL2 loop than GlyT1 as well as GAT1 and SIT1. Among these transporters, ATB^0,+^ possesses the longest EL2 loop overall. The EL4 of PROT and ATB^0,+^, which contains a helical V-shaped fragment, as well as their EL6 loops, are of similar length to those of GlyT1 and GAT1 [25,27]. In comparison to them, SIT1 and related neutral amino acid transporters have a significantly extended EL4 loop connected to an elongated TM7 [28,29]. PROT, ATB^0,+^, and GlyT1, like most SLC6 transporters, have an uninterrupted helical TM10, while GAT1 and other GABA transporters contain a non-helical insertion in this region [27,30]. In all transporters, TM1 and TM6 possess non-helical fragments in the middle of their length and, together with TM3 and TM8, they form the S1 binding site (Figure 1, left and right upper panels). Nearby, ion binding sites can be found. This overall arrangement was noticed in our PROT and ATB^0,+^ models as well as experimental structures of GlyT1, GAT1, and SIT1 transporters [25,27,31].

The transport mechanism of substrates across the biological membranes involves conformational changes in domains and amino acids. During the transport cycle, the protein adopts three main conformational states (Figure 1, down panel). First, in the outward-open state, the transporter faces the extracellular environment. Ligands can interact within the S2 site, which is a part of the extracellular vestibule. The residues forming the extracellular gate are distant from each other, allowing the binding of sodium and chloride ions to their respective sites, as well as substrates binding at the S1 site. The transporter then shifts to the occluded state, in which both extracellular and intracellular gates are closed. Substrates and ions are blocked inside the transporter. This state can be divided into outward-occluded and inward-occluded, depending on the direction the transporter is facing. Finally, in the inward-open state, the intracellular gate opens, the residues move apart, and the transporter releases substrate and ions into the cytoplasm, completing the cycle (Figure 1, down panel) [30]. PROT and ATB^0,+^ models in different conformational states are presented in Figure S1 while their binding sites are shown in Figure S2.

#### 2.2.2. Binding Site Residues

**Figure 2:**
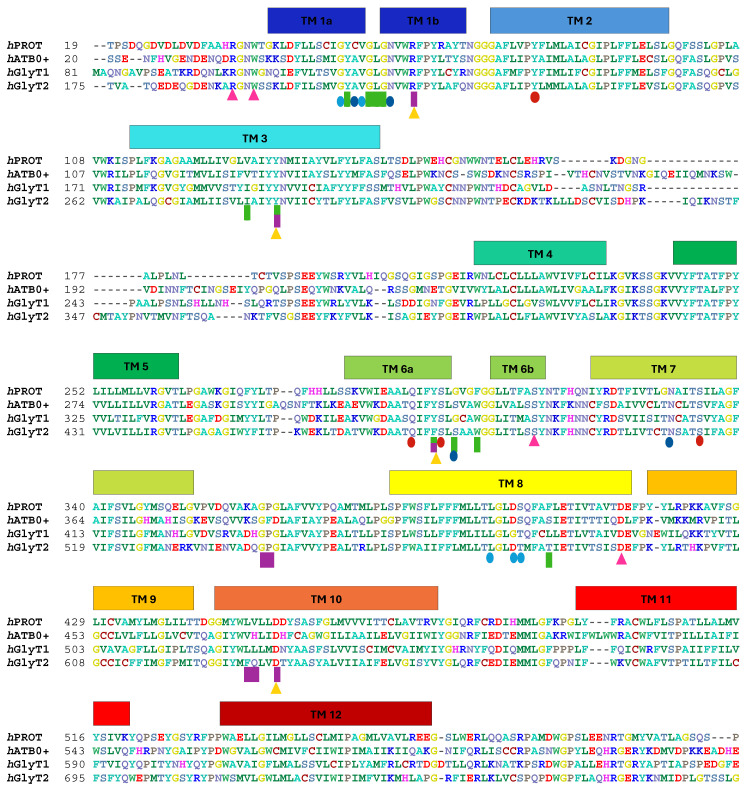
Alignment of the amino acid sequences of human glycine transporter subfamily. N- and C-terminus were omitted. Transmembrane domains are marked with distinct colours. Residues that form gates are marked with triangles: extracellular gate—yellow (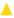), intracellular gate—pink (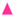). Residues involved in ion binding are marked with circles: Na^+^1 binding site—blue (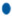), Na^+^2 binding site—light blue (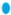) and Cl^−^ binding site—red (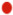). Potential residues for Na^+^3 site in GlyT2 or ATB^0+^ are not marked. Amino acids involved in ligand binding are marked with a rectangle: S2 site binding site—violet (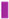), S1 binding site—green (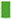).

To evaluate the structure of binding sites across transporters, we performed a sequence alignment, focusing particularly on residues involved in ligand recognition [32,33]. The structures of *a*LeuT, *d*DAT, and *h*SERT have been well-documented in the literature, providing a foundation for understanding similar transporter systems [34]. In our analysis, we compared key residues involved in ligand binding between PROT and ATB^0,+^ with those found in *h*GlyT1 [35] and *h*GlyT2 [36,37] (Figure 2).

Ligands and inhibitors initially interact with the S2 site. This site in PROT is formed by: Gly363, Pro364, Leu450, and Val451, as well as four residues from the extracellular gate, which define the bottom of the S2 site. Glycine is conserved among transporters, while the remaining three residues differ from one another: Pro364 (PROT) is replaced by Phe388 in ATB^0,+^, while Leu450 (PROT) is replaced by Phe629 in GlyT2. Val451 from PROT is substituted with His475, Leu525, and Gln630 in ATB^0,+^, GlyT1, and GlyT2, respectively.

**Figure 3:**
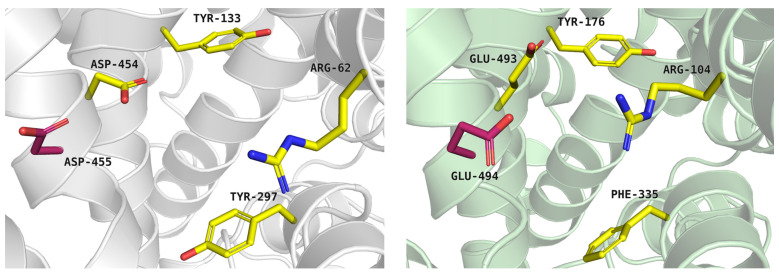
Extracellular gate of the proline transporter model, built on the template of dopamine transporter (PDB: 4XP9), tool: Modeller (left panel, grey cartoon), and serotonin transporter experimental structure—PDB code: 7TXT (right panel, green cartoon). Residues are coloured as follows: yellow—extracellular gate; residue marked in pink—Asp455 and Glu494.

The extracellular gate separates the S2 site from deeper S1 and ion sites, thereby regulating ligand access to the central binding core. In the glycine transporter subfamily, the extracellular gate is formed by two pairs of residues: arginine–aspartic acid and tyrosine–tyrosine (Arg62-Asp454, Tyr133-Tyr297 in PROT). In GlyT2, however, the second pair is composed of tyrosine–phenylalanine (Tyr287-Phe476). The importance of residues from extracellular gate has been proved in the past [38]. In PROT, a shift in Tyr133 may impair substrate transport, as proposed in patients with developmental delay caused by the Gly396Ser mutation. Gly396 is located close to Tyr133 from the extracellular gate, as well as residues from Na^+^1 binding site. Gly396Ser mutation showed reduced proline transport at a level of 30% of that of the WT protein and reduced cell-surface expression [12]. In PROT, Asp454 from extracellular gate is followed by Asp455 (Figure 3, left panel). It is possible that both residues contribute to ligand recognition. In another proline transporter, SIT1, Asn461 from extracellular gate is directly followed by Asp462. These residues have been suggested to restrict substrate access to the binding site [31]. A similar arrangement is found in *h*SERT, where the two following acid residues were observed: Glu493 and Glu494 (Figure 3, right panel). These acidic residues are proposed to take part in the initial binding of sodium ion. Movement of Glu493 may facilitate ion access to the binding site and subsequently support serotonin interaction at the S2 and S1 sites [39].

**Figure 4:**
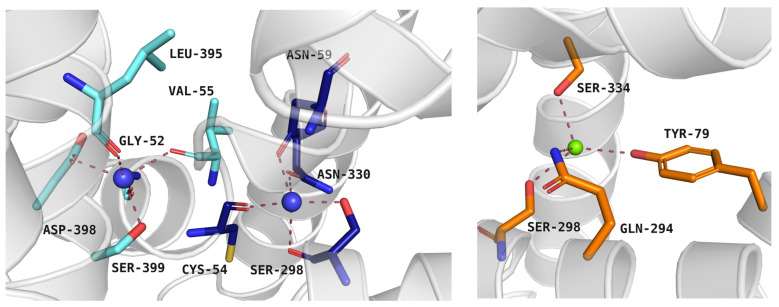
Residues from Na^+^1 and Na^+^2 binding sites (left panel) and Cl^−^ binding site (right panel) of the proline transporter model in the outward-occluded conformation (template: 4XPH, tool: SwissModel). Residues are coloured as follows: light blue—Na^+^2 binding site, dark blue—Na^+^1 binding site, orange—Cl^−^ binding site, blue spheres—sodium ions, green sphere—chloride ion.

At the beginning of transport, protein is open to the extracellular environment and is able to accept ions and ligands. PROT requires two Na^+^ ions and one Cl^−^ ion to transport one substrate molecule, similar to GlyT1, GAT1, and SIT1, whereas GlyT2 operates with a stoichiometry of three Na^+^ ions: one Cl^−^ ion: one substrate molecule. ATB^0,+^ is thought to operate two Na^+^ or even three Na^+^ ions: one Cl^−^ ion: one substrate molecule [40,41,42]. In PROT, the first Na^+^ ion binds to the Na^+^1 binding pocket formed by: Cys54, Asn59, Ser298, and Asn330. The second Na^+^ ion is coordinated by Gly52, Val55, Leu395, Asp398, and Ser399 in Na^+^2 binding site (Figure 4, left panel). The Cl^−^ ion binds between Tyr79, Gln294, Ser298, and Ser334 (Figure 4, right panel). Residues coordinating the ions are mostly conserved across the entire SLC6 family, although some variations have been observed. Compared to PROT, ATB^0,+^ and GlyT1 have a small, alanine residue in place of Cys54 in Na^+^1 site. Additionally, Ser399 from Na^+^2 site in PROT is replaced by Thr472 in GlyT1 and Thr582 in GlyT2. GlyT2 uses three sodium ions for substrate transport, although the exact localization of the Na^+^3 binding site remains under discussion [36,43].

In the proline transporter, the S1 site is formed by: Tyr53, Gly56, Leu57, and Gly58—residues conserved across the glycine subfamily—as well as Val129, Gly300, Phe303, and Phe403, which vary between proteins (Table 2). Additional residues contributing to the S1 site include Tyr133 and Tyr297 from the extracellular gate, and Cys54, Ser298 from the ion binding site. Val129 in PROT and Val128 in ATB^0,+^ correspond to Ile192 in GlyT1 and Ile283 in GlyT2. The role of Val128 in ATB^0,+^ has been evaluated through mutagenesis [44]. The Val128Phe mutant showed a loss of transport for arginine and lysine [44], while the V128I mutant had no significant effect on transporter activity [44]. Another difference between proteins is the presence of Gly300 in PROT and Gly373 in GlyT1, whereas in ATB^0,+^ and GlyT2, this position is occupied by Ser324 and Ser479, respectively. PROT possesses two aromatic residues: Phe303 and Phe403 (Table 2). Phe403 in PROT is substituted by Ser427 in ATB^0,+^, Leu476 in GlyT1, and Thr582 in GlyT2. Phe303 in PROT is replaced by tryptophan in ATB^0,+^ (Trp327), GlyT1 (Trp376), and GlyT2 (Trp482). Mutation at this residue in GlyT2 enabled the transport of different amino acids [45]. Tryptophan may change orientation of its side chain, facilitating substrate recognition [46].

In the occluded conformation, residues from both the extracellular and intracellular gates are positioned close to each other. When the transporter shifts to the inward-open state, the interactions between intracellular gate residue pairs: arginine–aspartic acid and tryptophan–serine, become disrupted (Table 2). This conformational change allows ligands and ions to be released into the cytoplasm.

### 2.3. Docking Studies and Molecular Dynamics Simulation

Docking studies enabled us to elucidate the binding modes of the substrates within the PROT and ATB^0,+^ transporters. Using their models, the structure of GlyT1 (PDB code: 8WFJ) and literature data on the glycine transporter subfamily, we compared protein–substrate interactions and identified key residues involved in ligand binding. Ligands were docked into homology models selected based on DOPEScore and QMEAN assessments. Additionally, selected inhibitors were docked into the transporters, and their binding modes were further evaluated through molecular dynamics simulations.

#### 2.3.1. Binding Mode of Substrates to Glycine Transporter Subfamily

Substrates of the glycine transporter subfamily (PROT, ATB^0,+^, GlyT1, GlyT2) share a common core, with an amino group and a carboxyl group linked by one carbon but differ in the side-chain features (Table 3). Proline, the primary substrate of “brain-specific” PROT, features a distinctive pyrrolidine ring with a secondary amine group [3]. Glycine, the main substrate of GlyT1 and GlyT2, is the simplest amino acid, consisting of just a single carbon, the amine and carboxyl group [35]. ATB^0,+^, in contrast, accommodates a wide range of neutral and cationic amino acids. Its highest affinity is observed for predominantly hydrophobic and branched-chain amino acids, such as isoleucine, leucine, valine, which feature aliphatic side chains; sulphur-containing methionine; and residues which possess aromatic side chains like phenylalanine, tyrosine, and tryptophan. The protein also transports small, neutral amino acids like alanine, glycine, and polar residues: serine and threonine, as well as cationic amino acids: histidine, lysine, and arginine, with their longer chains and charged functional groups. The ATB^0,+^ has potentially physiologically irrelevant affinity for the proline [5,6]. Transporter accepts L-amino acids, which take part in metabolic pathways in mammals, although it has been suggested that some D-amino acids might also be transported [47]. This broad substrate range highlight the functional versatility of ATB^0,+^, compared to the stricter preferences of PROT and GlyTs.

**Figure 5:**
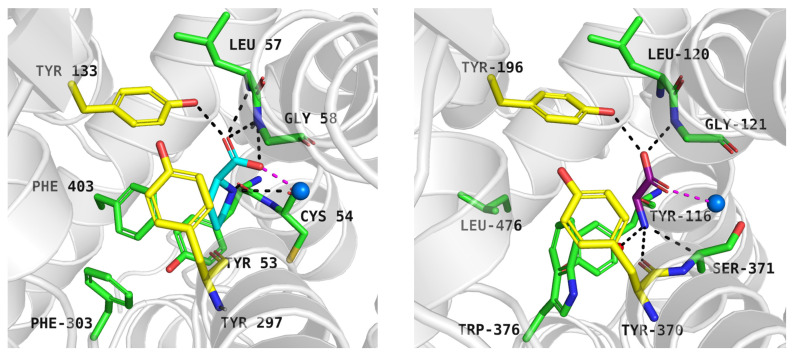
Proline binding mode within PROT model in inward-occluded state—left panel (template: 7Y7W, tool: Modeller) and glycine within GlyT1 experimental structure in outward-occluded state (PDB code: 8WFI)—right panel. Residues are coloured as follows: yellow—extracellular gate, green—S1 site, blue sphere—sodium ion. Proline—blue, glycine—purple.

**Figure 6:**
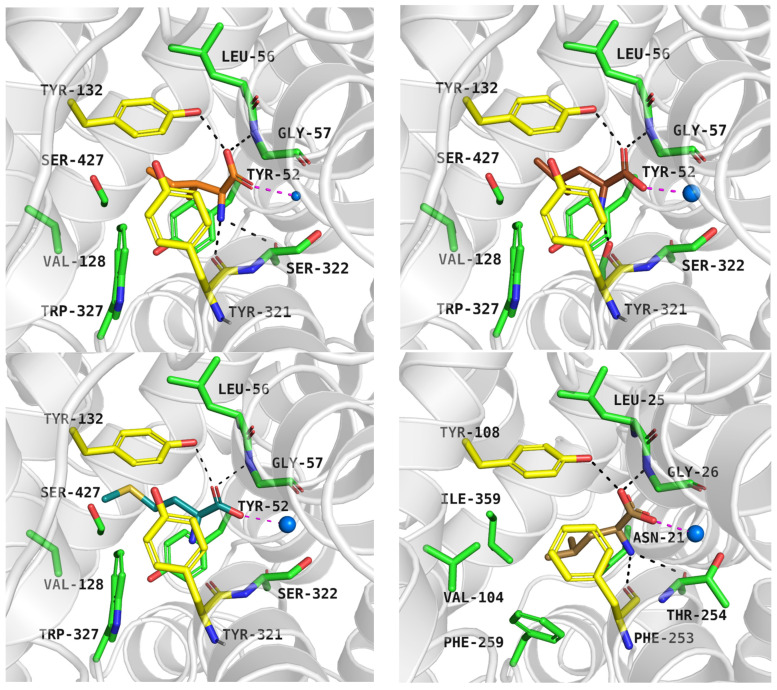
Substrate binding mode within the ATB^0,+^ model in the inward-occluded state (template: 7Y7W, tool: Modeller): isoleucine—left, upper panel, leucine—right, upper panel, methionine—left, down panel. The right, down panel shows the binding mode of leucine within bacterial analogue *a*LeuT (PDB code: 2A65). Residues are coloured as follows: yellow—extracellular gate, green—S1 site, blue sphere—sodium ion, isoleucine—orange, leucine—brown, methionine—green.

To understand substrate preferences across the glycine transporter subfamily, we investigated substrate binding modes, starting from proline and PROT. Due to the large number of ATB^0,+^ substrates, selected representatives from neutral and cationic group were extensively discussed, including amino acids with the highest affinity for the transporter. So far, there have been no studies that describe proline binding to PROT, while substrates of ATB^0,+^ have been evaluated only in the outward-open conformation [48]. In the present work, we generated homology models of both transporters in four conformational states and, for the first time, provide an in-depth analysis of substrate binding, focusing on the inward-occluded state of the transporters. This closed conformation, represented by one of the best-scoring models built on the *h*GAT1 template (PDB code: 7Y7W), likely supports tighter substrate binding mode than the outward- or inward-open states. Binding mode of proline was compared with the experimentally resolved complex of *h*GlyT1 with glycine (PDB code: 8WFI) [35]. Additionally, for ATB^0,+^, the complex of *a*LeuT with leucine was included for analysis (PDB code: 2A65) [49]. The ligands adopted similar orientations across all transporters (Figure 5 and Figure 6).

Proline, docked to PROT model in the inward-occluded state, received a docking score of −7.6 kcal/mol. It was predicted to interact with PROT through its carboxyl group, positioned near Tyr133, Gly58, and sodium ion, in a manner similar to the one observed in experimentally resolved GlyT1-glycine complex (Figure 5, left and right panels). The amine group of the cyclic ring formed a cation-π interaction with the aromatic ring of Tyr53, Tyr133, and a hydrogen bond with the main chain of Cys54 (Figure 5, left panel). Notably, the binding site of PROT adopted space for cyclic ring of proline through the arrangement of aromatic residues from extracellular gate (Tyr133, Tyr297) and S1 binding site (Phe303, Tyr53, and Phe403) (Figure 5, left panel). Phe403, which is absent in the equivalent position in other glycine transporter subfamily members, may contribute to the selective recognition of proline.

Neutral substrates docked to the ATB^0,+^ binding site obtained the following docking scores: isoleucine −7.1 kcal/mol, leucine −6.7 kcal/mol, and methionine −5.6 kcal/mol. These values are consistent with their decreasing affinity for the transporter, reflected by EC_50_ = 6 µM, EC_50_ = 12 µM, and EC_50_ = 14 µM, respectively [6]. Their main interactions via the carboxy and basic groups (Figure 6, upper panels, left, down panel) were analogous to those in proline in PROT and glycine in GlyT1 (Figure 5, left and right panels). Additionally, leucine in ATB^0,+^ adopted a binding mode similar to that observed in the crystal structure of leucine in the bacterial analogue *a*LeuT (Figure 6: ATB^0,+^—right, upper panel, *a*LeuT—right, down panel) [49].

The carboxy group of the ligands interacted with Tyr132, Gly57, and sodium ion, while their amine groups formed hydrogen bonds with the main chain of Tyr321 (Figure 6). The aliphatic side chain of the neutral substrate leucine interacted with a hydrophobic pocket of ATB^0,+^ composed of Tyr52, Val128, Tyr132, Tyr321, and Trp327, in a manner similar to that observed in *a*LeuT. The Tyr52 in ATB^0,+^ is replaced by Asn21 in the case of *a*LeuT, which could affect the ligand’s orientation (compare Figure 6, right upper and right down panels). Residues Tyr52 and Trp327 from S1 binding site as well as Tyr132 and Tyr321 from extracellular gate take part in hydrophobic interactions with side chain of neutral ligands: isoleucine, leucine, methionine, and form cation-π interactions with their basic main chains (Figure 6, upper panels, and left, down panel).

**Figure 7:**
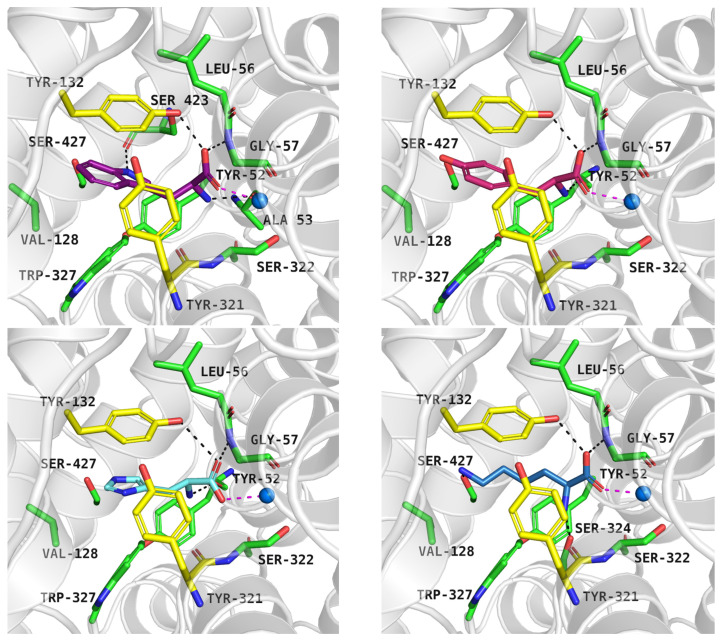
Substrate binding mode within the ATB^0,+^ model in the inward-occluded state (template: 7Y7W, tool: Modeller): tryptophan—left, upper panel, tyrosine—right, upper panel, histidine—left, down panel, lysine—right, down panel. Residues are coloured as follows: yellow—extracellular gate, green—S1 site, blue sphere—sodium ion, tryptophan—violet, tyrosine—pink, histidine—light blue, lysine—blue.

Trp327 located at the S1 binding site in ATB^0,+^ may facilitate ligand accommodation through rotation of its indole-containing side chain. Docking to the rigid protein in the inward-occluded state showed that phenylalanine and tryptophan could not bind within the S1 site. Allowing Trp327 to rotate in the Induced Fit Docking (IFD) module improved ligand binding and scores, as observed for aromatic substrates: tryptophan (−9.0 kcal/mol) and tyrosine (−8.7 kcal/mol) (Figure 7, left and right upper panel); as well as charged substrates: histidine (−6.8 kcal/mol) and lysine (−7.0 kcal/mol) (Figure 7, left and right down panel). The corresponding affinities for the transporter are as follows: EC_50_ = 2, EC_50_ = 92 µM, EC_50_ = 76 µM, EC_50_ = 100 µM [6]. Aromatic amino acids formed ring interactions with Tyr132 from the extracellular gate and Trp327 from the S1 active site. In GlyT2, mutation of the analogous residue, Trp482, enabled the transport of alternative substrates such as alanine, or resulted in the loss of glycine transport [45]; however, similar studies have not yet been conducted for ATB^0,+^.

**Figure 8:**
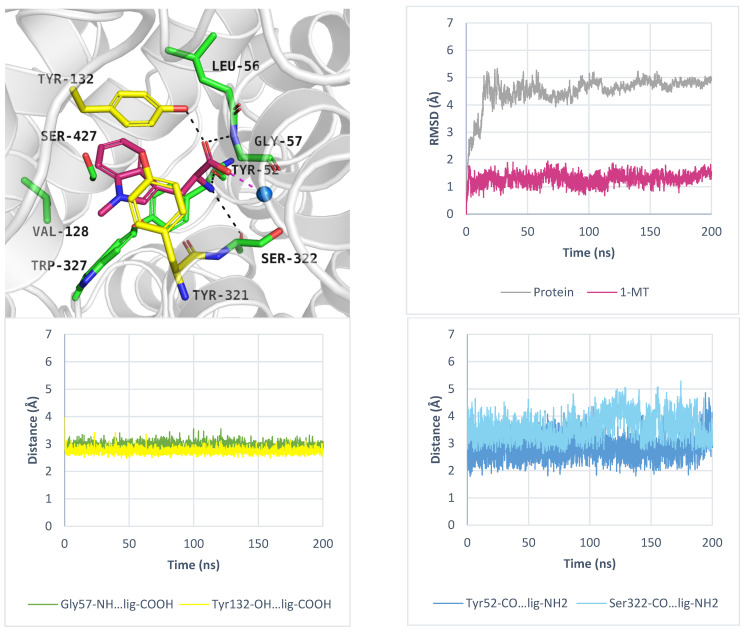
Binding mode of 1-MT within ATB^0,+^ model in inward-occluded state, template: 7Y7W, tool: Modeller (left upper panel). Residues are coloured as follows: yellow—extracellular gate, green—S1 site, blue sphere—sodium ion, 1-MT—pink. RMSD changes in protein and 1-MT during molecular dynamics (right, upper panel) and distance changes between 1-MT and amino acids (left and right down panels). Interactions are coloured as follows: green; Gly57-NH—ligand, yellow; Tyr132-OH—ligand, blue; Tyr52-CO—ligand, light blue; Ser322-CO—ligand.

As mentioned, ATB^0,+^ can transport analogues of proteinogenic amino acids, among them 1-methyltryptophan (1-MT). During IFD, the ligand received a docking score of −8.9 kcal/mol. Typical interactions with the sodium ion and key residues were preserved. The indole moiety of the compound formed aromatic interactions with Tyr132, Tyr321, and Trp327, while methyl substituent of 1-MT was located near Val128 and Trp327 (Figure 8, left, upper panel). Molecular dynamics simulations confirmed that the inhibitor binding mode predicted by docking studies was accurate. The average RMSD values for the protein and the inhibitor were 4.5 Å and 1.3 Å, respectively. Hydrogen bonds between 1-MT and Gly57, Tyr132 were the most stable (Figure 8, left, down panel), whereas interactions with Tyr52 and Ser322 were weak or missing (Figure 8, right, down panel). The ligand RMSD changes over three independent 200 ns molecular dynamics simulations are shown in Figure S5.

#### 2.3.2. Docking of Inhibitors of PROT and ATB^0,+^ Transporters

Various small-molecule inhibitors of glycine transporter (GlyT1, GlyT2) have been identified, demonstrating promising activity in modulating transporter function [50]. However, the pool of known ligands for PROT and ATB^0,+^ is limited. Experimental studies have shown that several ligands bind to the structures of GlyT1 from the intracellular side [25,35]. Nevertheless, it is likely that some inhibitors may also bind from the extracellular side [50], as observed in other members of the SLC6 transporter family. The binding mode of some PROT inhibitors has been investigated in the inward open state [9], while ligands of ATB^0,+^ were studied in the outward open state [48]. Still, the precise modes of inhibitor binding remain unclear.

Inhibitors of the proline transporter are small molecules with various potencies, ranging from micromolar to nanomolar concentrations [9,10,51,52]. Most PROT inhibitors share an aryl piperazine scaffold (e.g., compound LQFM215, Table 4 and Table S3) [9]. Notably, these compounds generally lack an acidic group and do not mimic the transporter’s natural substrate, proline. Two compounds, LP403812 (Table 4, middle panel) and B52, contain a pyrazolo-thiazole core (Table S3) [51,53]. In contrast, the inhibitor of the ATB^0,+^ transporter, α-methyltryptophan (α-MT), mimics its natural substrate, tryptophan, featuring both an amine and an acidic group (Table 4, right panel) [54].

Our docking studies to the PROT transporter revealed that inhibitors fit into the outward-open and inward-open states. In contrast, they could not be accommodated in either the outward-occluded or inward-occluded conformations. No docking results were obtained for these states (Table S4), while proline was successfully docked. This can be explained by the substantially larger volume and steric bulk of the inhibitors compared to the natural substrate, as well as by the reduced cavity size in the occluded states caused by Tyr132 and Tyr321 in the extracellular gate together with residues forming the S1 site. This observation may suggest that the PROT inhibitors could sterically block the transition of the transporter toward an occluded state during the transport cycle, although this hypothesis requires experimental verification.

**Figure 9:**
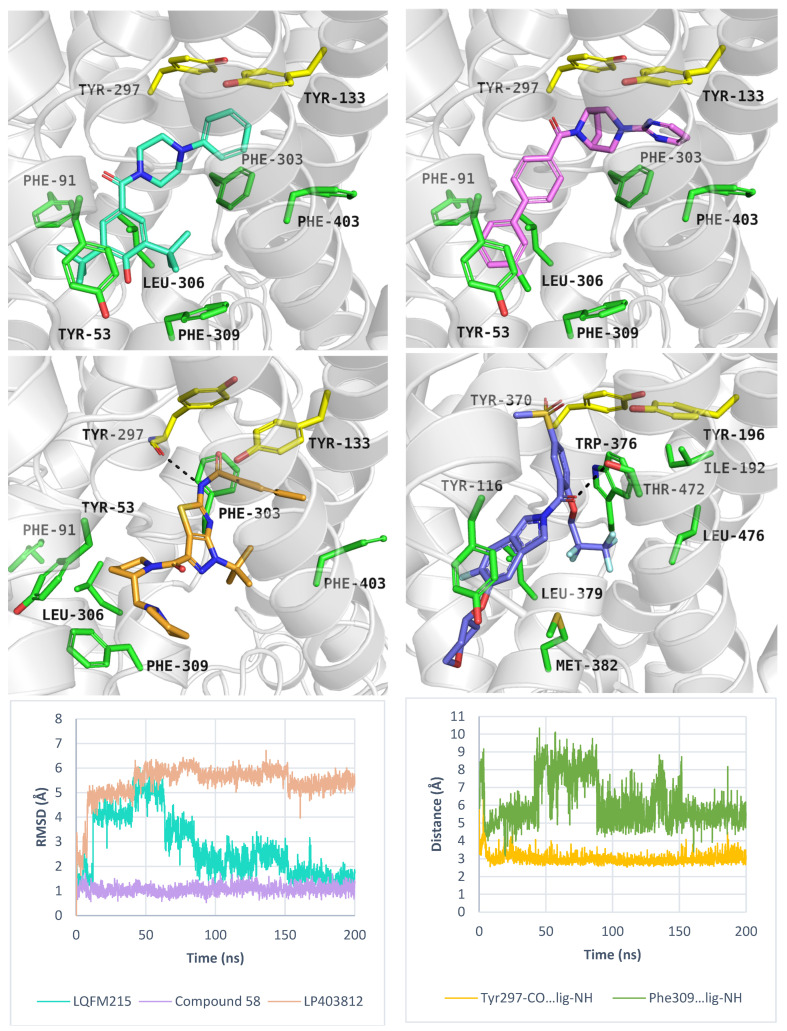
Binding mode of inhibitors within PROT model in the inward-open state, template: 6ZBV, tool: Modeller: LQFM215 (left, upper panel), compound 58 (right, upper panel), LP403812 (left, middle panel). Experimental GlyT1 complex with “Cmpd1” (PDB code: 6ZBV), (right, middle panel). RMSD changes in inhibitors during molecular dynamics (left, down panel), and distance changes between Tyr297-CO, Phe309-Ar and LP403812 (right, down panel). Residues are coloured as follows: yellow—extracellular gate, green—S1 site, blue sphere—sodium ion, compound LQFM215—cyan, 58—violet, LP403812—sand, Cmpd 1—blue.

Overall, the best docking scores were observed in the inward-open state, rather than the outward-open state (Table S4). The inhibitor LQFM215 (IC_50_ = 20.4 µM), received a docking score of −8.4 kcal/mol in the inward open state. Its binding mode was similar to that of another compound with an aryl piperazine scaffold. Among this group, the most active PROT inhibitor—ligand 58 (IC_50_ = 18 nM) received the docking score of −9.4 kcal/mol, which was one of the highest results among all docked ligands (Table S4). Inhibitor LP403812 (IC_50_ = 0.11 nM) scored −7.0 kcal/mol. Figure 9 presents their binding mode within PROT in inward-open state and experimental structure of GlyT1 with “Cmpd1” (upper and middle panels). The ligands interacted with PROT through hydrophobic interactions involving aromatic residues such as Tyr53, Tyr133, Tyr297, Phe303, Phe309, and Phe403. It was observed that tested compounds did not form hydrogen bonds with residues from the extracellular gate or S1 binding site, particularly Tyr133 or Gly57. For selected inhibitors: LQFM215, 58 and LP403812, we performed MM-GBSA calculations to estimate their binding free energies. In all cases, the calculated free energies were lower in the inward-open state than in the outward-open conformation, suggesting more favourable binding. The estimated ΔG_bind_ values were as follows: LQFM215: –25.33 kcal/mol; compound 58: –24.87 kcal/mol; LP403812: –51.89 kcal/mol. To further evaluate the stability of predicted binding poses, molecular dynamics simulations were conducted. Ligand LQFM215 adopted stable pose, with RMSD value of 1.5 Å after 150 ns of simulation (average RMSD = 2.8 Å). Compound 58 showed the best binding mode, with the lowest average RMSD value 1.0 Å, while LP403812 displayed the highest RMSD value of 3.9 Å (Figure 9, left, down panel). All ligands remained within the binding site through the entire 200 ns simulation period across three independent replicates. Their RMSD values in each run are presented in Figure S6. Interestingly, during molecular dynamics, the ligand LP403812 was able to form a hydrogen bond interaction with the main chain of Tyr297 from extracellular gate. Additionally, a cation-π interaction appeared between protonated nitrogen from the pyrrolidine ring and aromatic ring of Phe309 (Figure 9, right, down panel).

**Figure 10:**
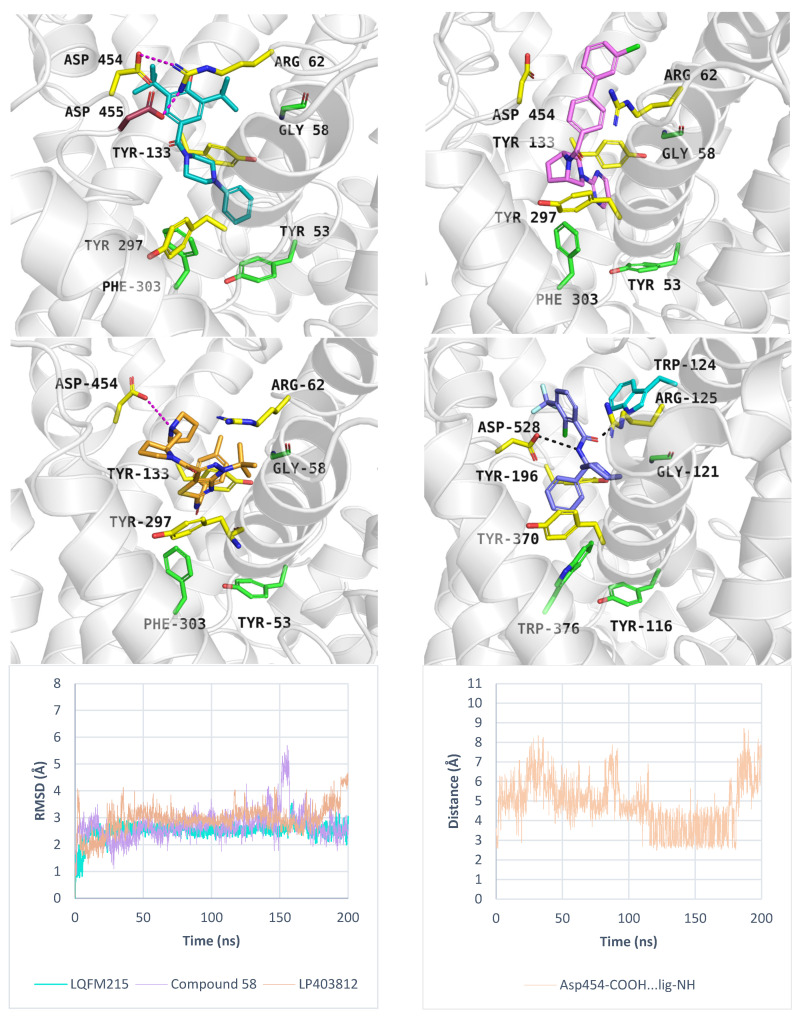
Binding mode of inhibitors within PROT model in the outward-open state, template: 4XP9, tool: Modeller—LQFM215 (left, upper panel) and compound 58 (right, upper panel), LP403812 (left, middle panel). Inhibitor SSR504734—GlyT1 complex (PDB code: 8WFK), (right, middle panel). RMSD changes in inhibitors during molecular dynamics (left, down panel), and distance changes between Asp454 and LP403812 (right, down panel). Residues are coloured as follows: yellow—extracellular gate, green—S1 site, blue sphere—sodium ion, compound LQFM215—cyan, 58—violet, LP403812—sand, SSR504734—blue.

Additionally, we performed molecular dynamics simulations for LQFM215, 58, and LP403812 bound to PROT in the outward-open state. These compounds showed less favourable docking scores (−5.0 kcal/mol, −5.6 kcal/mol, −4.8 kcal/mol, respectively) and MM-GBSA ΔG_bind_ values (11.43 kcal/mol, −6.85 kcal/mol, −21.94 kcal/mol, respectively), compared to the inward-open state. Molecular dynamics simulations showed that LQFM215 and LP30812 adopted stable poses, with RMSD value of 2.5 Å, and 3.0 Å, respectively. The most active compound 58 showed an RMSD value of 2.7 Å, which was higher than that in the best run for the inward-open state. Figure S7 presents the RMSD values of the inhibitors in each MD run. In our models, the tested ligands were located below EL4 and interacted with Arg62 or Asp454 from the extracellular gate (Figure 10). The inhibitor LQFM215 was predicted to form cation-π interactions with Arg62, while this residue also engaged in ionic contacts with Asp454 and Asp455. These interactions may contribute to the stabilization of the inhibitor within the transporter (Figure 10, left, upper panel). Compound 58 exhibited a similar orientation within the PROT binding site. Its nitrogen-containing moiety was located between Tyr133 and Tyr297, while its bulky, aromatic fragment was located near Arg62 (Figure 10, right, upper panel). Ligand LP403812 was able to form an ionic interaction between Asp454 and the protonated nitrogen of the pyrrolidine ring (Figure 10, left, middle panel; right, down panel). This moiety may mimic proline and its binding mode. However, the absence of an acidic group likely impaired stable binding by preventing interactions with key residues from the S1 site (Gly58) and extracellular gate (Tyr133). Similar ionic interactions involving an aspartic acid residue from the extracellular gate have been reported for GlyT1 in complex with SSR504734 [35] (Figure 10, right, middle panel).

Molecular dynamics simulation showed that tested ligands adopted beneficial binding modes in both outward- as well as inward-open states. However, PROT inhibitors docked in the inward-open state received better docking scores and estimated free energy, compared to the outward open state. Inhibitors formed predominantly hydrophobic interactions with aromatic residues such as Tyr53, Tyr297, Phe303, and Phe403. In case of LP403812, hydrogen bond with Tyr297 from extracellular gate, as well as cation-π interaction with Phe309 were observed. However, our in silico predictions using homology models need experimental validation. Given the similarities between the binding sites of PROT and GlyT1, and the interactions predicted between tested inhibitors and conserved residues within the glycine transporter subfamily, we suggest that it is possible that LQFM215, compound 58, LP403812 or other PROT inhibitors might also bind to GlyT1 or GlyT2. However, data on their selectivity between PROT and GlyT1/GlyT2 remain limited. LP403812 has been shown to inhibit PROT activity (IC_50_ = 110 nM), with no effect on GlyT1 and DAT transporter activity in concentrations up 10 µM [51]. To explore potential cross-activity, we performed additional docking studies using GlyT1 in an inward-open state (PDB code: 6ZBV). Almost all tested ligands received less beneficial docking scores within GlyT1 in comparison to the PROT (Table S4). Compound LP403812 received docking score in value −4.84 kcal/mol, which is consistent with its reported lack of activity against GlyT1 [51]. Interestingly, bitopertin, a potent GlyT1 inhibitor (IC_50_ = 25 nM), has been proposed based on the in silico studies to potentially inhibit PROT as well [55]. In our docking simulations, bitopertin also received a favourable docking score to PROT (−8.02 kcal/mol), supporting this hypothesis. Further experimental validation is needed to confirm this potential cross-reactivity within the glycine transporter subfamily and their binding mode.

**Figure 11:**
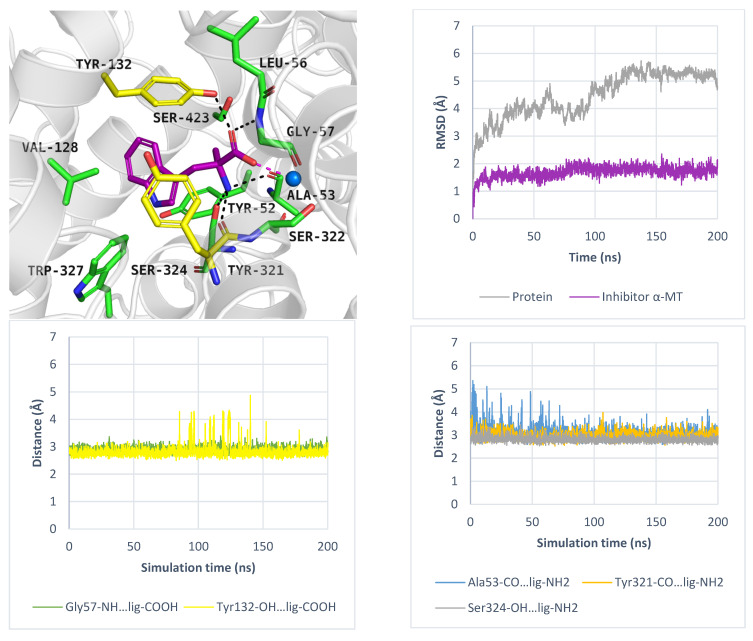
Binding mode of inhibitor α-MT within ATB^0,+^ model in the inward-occluded state, template: 6ZBV, tool: Modeller (left upper panel). Residues are coloured as follows: yellow—extracellular gate, green—S1 site, blue sphere—sodium ion, α-MT—violet. RMSD changes in ATB^0,+^ and α-MT (right, upper panel). Distance changes between α-MT and residues (left and right down panels). Interactions are coloured as follows: green; Gly57-NH—ligand, yellow; Tyr132-OH—ligand, blue; Ala53-CO—ligand, orange; Tyr321-CO—ligand, grey; Ser324-CO—ligand.

While PROT or GlyT1 transporter inhibitors have biological activity in the nanomolar range, the known ATB^0,+^ inhibitor: α-methyltryptophan (α-MT) displays an IC_50_ = 250 µM [54]. This compound, which mimics the natural substrate tryptophan, blocks the transporter, promotes autophagy, and inhibits the proliferation of cancer cells [54]. In induced-fit docking, the ligand received a docking score of −9.7 kcal/mol, and its binding mode was further evaluated using molecular dynamics simulations. The results are shown in Figure 11. The average RMSD values for the protein and inhibitor were 4.4 Å and 1.7 Å, respectively (Figure 11, right, upper panel). The inhibitor RMSD changes over three independent 200 ns molecular dynamics simulations are shown in Figure S8. Key interactions with sodium ion and residues such as Tyr132 and Gly57 were maintained (Figure 11, left, down panel). Additionally, specific hydrogen bonds were formed with Ala53 and Tyr321. All interactions remained stable during MD. A transient hydrogen bond with Ser324 was also observed (Figure 11, right, down panel). These specific interactions, compared to those formed by the substrates: tryptophan and 1-MT, may determine inhibitory activity of α-MT, although experimental validation is needed. Moreover, the indole moiety of compound created aromatic interactions with Val128, Tyr132, and Tyr321, while the methyl substituent of α-MT was located near Ser423 and Tyr132. Given the increasing recognition of the role of ATB^0,+^ in cancer development and progression, there is a growing need to identify new ligands for this transporter, which could be tested as anticancer agents.

This study relies exclusively on computational modelling since no experimental structures of PROT or ATB^0,+^ are available. The predicted conformational states therefore depend on the quality and sequence similarity of the selected templates. Binding poses obtained from docking and molecular dynamics simulations reflect energetically favourable configurations within the models, but the results require experimental confirmation. Additionally, the simulations were performed without incorporating the Na^+^ electrochemical gradient that physiologically drives the transport process, and they do not capture dynamic protonation changes in residues in the binding sites or substrates that may influence binding affinity and conformational transitions during the transport cycle. These methodological constraints should be considered when interpreting the results, and future biochemical and structural studies will be essential to verify the proposed mechanisms and ligand interactions.

## 3. Materials and Methods

### 3.1. Homology Modelling

Amino acid sequences of SLC6 family transporters were retrieved in FASTA format from the UniProt database (The UniProt Consortium, Hinxton, UK) [56] and aligned using Clustal Omega (EMBL–EBI, Hinxton, UK) [57]. Templates selection for homology modelling considered factors such as resolution, sequence mutations, and conformational states. Consequently, 14 experimental structures were chosen, including: *Aquifex aeolicus* LeuT (*a*LeuT) in the outward-occluded state (PDB codes: 2A65, 2Q72, 2Q6H), and outward-open state (PDB codes: 3FAF, 4MMB, 4MM7); *Drosophila melanogaster DAT* (*d*DAT) in the outward-open state (PDB codes: 4XP4, 4XP9 6M2R), and outward-occluded state (PDB code: 4XPH); human SERT (*h*SERT) in the outward-open state (PDB code: 5I6X) and human GlyT1 (*h*GlyT1) in the inward-open state (PDB code: 6ZBV). These structures were resolved by X-ray diffraction, with resolutions ranging from 1.65 to 3.40 Å. Additionally, *h*GAT1 structures in the inward-open state (PDB code: 7Y7Z) and inward-occluded state (PDB code: 7Y7W)[27] by cryogenic electron microscopy were used (Table S1). Sequence alignment of the templates and PROT and ATB^0,+^ was performed using BioEdit [58], excluding the N- and C-termini due to low sequence similarity. Homology modelling was carried out using both the SwissModel server (https://swissmodel.expasy.org, accessed on 4 December 2024). and the Modeller 9.15 software (University of California, San Francisco, CA, USA). SwissModel was used to generated one model per template, while Modeller generated 200 models per template using the MyModel class. The resulting models were assessed using DOPE and QMEAN scores. The top models were selected for subsequent docking studies, and their evaluation is summarized in Table S2. Sodium and chloride ions from the template structures were retained in the models whenever possible. If ions were missing, the models were superimposed onto ion-containing templates, and the ions were subsequently incorporated into the models.

### 3.2. Docking Studies

The ligands were prepared using the Maestro software (Schrodinger Suite 2023-4, Schrödinger Inc., New York, NY, USA). Ionization of the compounds was carried out at a physiological pH of 7.4 ± 0.2, using the Epik function within the LigPrep module. Prior to docking, proteins underwent processing with the Protein Preparation Wizard under default parameters at pH 7.4 ± 0.2, including optimizing hydrogen bonds and applying restrained minimization. The docking grid was defined with an inner box of dimensions 15 Å × 15 Å × 15 Å and an outer box of 25 Å × 25 Å × 25 Å. Docking simulations were executed in standard precision, generating 10 poses per ligand, which were ranked by their Docking Score. The OPLS4 force field was employed throughout the study. For docking to the inward-occluded state of ATB^0,+^, the Induced Fit Docking module was employed, allowing for Trp327 flexibility. Docking results were analyzed using Maestro and PyMOL v3.1 (Schrödinger, LLC, New York, NY, USA).

### 3.3. MM-GBSA Calculation

MM-GBSA calculations were performed using Prime v6.1 software (Schrödinger Inc., New York, NY, USA) to estimate the binding affinities of selected inhibitors. The top-scoring binding pose from the glide docking was used for each inhibitor. To reflect physiological conditions, ion positions were preserved, and residues surrounding the ligand were kept rigid. Calculations were carried out using the VSGB solvation model, with default settings for all other parameters.

### 3.4. Molecular Dynamics Simulations

For molecular dynamics simulations, we utilized the Desmond 6.3 software (Schrödinger Inc., New York, NY, USA). Initially, the transporter models were embedded into the membrane through the OPM server [59]. The simulation protocol in Desmond involved preparing the input files using the System Builder module, with the TIP3P water model and a POPC membrane. The transporter model, pre-aligned via the OPM server, was used, and an orthorhombic box was subsequently defined using the buffer method. Chloride ions were added to neutralize the system, and 0.15 M NaCl was incorporated to simulate physiological conditions. Following system minimization, all-atom molecular dynamics (MD) simulations were run under NPT conditions for 200 ns, with data recorded every 200 ps. The simulations were performed using the OPLS3e force field. Each transporter–ligand complex was subjected to three independent simulation runs, using a random seed. Trajectory analyses (RMSD and ligand–residue distance monitoring) were carried out in Maestro using the Simulation Interaction Diagram and Trajectory Analysis tools.

## 4. Conclusions

In this study we characterized the structural properties of proline transporter (PROT, SLC6A7) and the neutral and cationic amino acid transporter (ATB^0,+^, SLC6A14) using a combination of in silico approaches, including homology modelling, docking studies, and molecular dynamics simulations. Homology modelling enabled the construction of PROT and ATB^0,+^ models in all conformational states: outward-open, outward-occluded, inward-occluded, and inward-open states, which were subsequently used in docking studies of substrates and inhibitors. Both transporters exhibited the same overall fold as other SLC6 family members and shared conserved amino acids in regions crucial for ligand recognition. We highlighted residues involved in ligand binding: Gly57, and Tyr133 (PROT numeration) and sodium ions, as well as in PROT, Phe403 while in ATB^0,+^, Trp327 and Tyr321. Comparative analysis of conformational states revealed that inhibitor binding and stability depend on both ligand type and transporter conformation. The models developed in this study provide a basis for designing novel ligands that could be tested in various assays to explore their potential applications in neurological disorders (PROT ligands) or their anticancer activity (ATB^0,+^ ligands).

## Figures and Tables

**Table 1:** Sequence alignment for members of the glycine subfamily and selected proteins with known structures.

Sequences	*d*DAT		*a*LeuT		*h*SERT		*h*GlyT1		*h*GAT1		*h*SIT1	
Identity (I.), Similarity (S.)	I. [%]	S. [%]	I. [%]	S. [%]	I. [%]	S. [%]	I. [%]	S. [%]	I. [%]	S. [%]	I. [%]	S. [%]
*h*PROT	41.7	58.3	22.1	36.8	36.2	51.5	**43.1**	60.2	**40.4**	58.4	29.4	43.4
*h*ATB^0,+^	**40.1**	55.9	19.8	34.2	36.0	53.6	37.1	54.1	**38.5**	55.8	29.7	44.8
*h*GlyT1	35.3	51.0	17.5	30.3	34.7	53.2	100.0	100.0	53.2	36.4	27.7	40.9
*h*GlyT2	33.9	47.8	18.4	30.3	33.2	48.6	38.9	55.1	33.9	48.2	25.9	40.7

*d*DAT—*Drosophila melanogaster* DAT, *a*LeuT—*Aquifex aeolicus* LeuT, *h*SERT—human SERT, *h*GlyT1*—*human GlyT1. Numbers in bold indicate the highest identity values for the compared sequences.

**Table 2:** Residues forming the S1 binding site and intracellular gate in selected transporters from the glycine transporter subfamily, as well as bacterial homologue, *a*LeuT.

Protein	S1 Binding Site								Intracellular Gate			
*h*PROT	Tyr53	Gly56	Leu57	Gly58	Val129	Gly300	Phe303	Phe403	Arg37	Trp40	Ser311	Asp413
*h*ATB^0,+^	Tyr52	Gly55	Leu56	Gly57	Val128	Ser324	Trp327	Ser427	Arg36	Trp39	Ser322	Asp437
*h*GlyT1	Tyr116	Gly119	Leu120	Gly121	Ile192	Gly373	Trp376	Leu476	Arg100	Trp103	Ser384	Asp486
*h*GlyT2	Tyr207	Gly210	Leu211	Gly212	Ile283	Ser479	Trp482	Thr582	Arg191	Trp194	Ser490	Asp592
*a*LeuT	Asn21	Gly24	Leu25	Gly26	Val104	Ser256	Phe259	Ile359	Arg5	Trp8	Asn361	Asp369

**Table 3:** Representative substrates of the glycine transporter subfamily: PROT, GlyT1, GlyT2, ATB^0,+^.

PROT	GlyT1	GlyT2
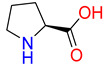	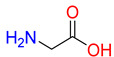	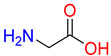
Proline	Glycine	Glycine
	**ATB^0,+^**	
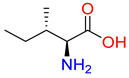	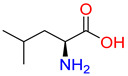	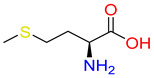
Isoleucine	Leucine	Methionine
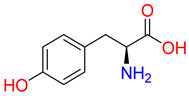	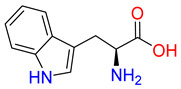	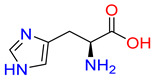
Tyrosine	Tryptophan	Histidine
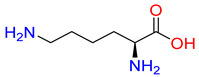	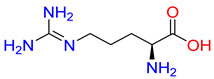	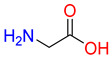
Lysine	Arginine	Glycine

**Table 4:** Selected inhibitors of PROT and ATB^0,+^ from the glycine transporter subfamily.

PROT		ATB^0,+^
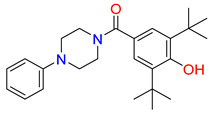	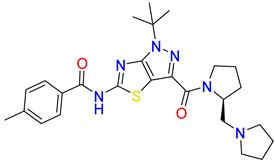	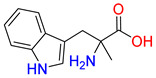
LQFM215, IC_50_ = 20.4 µM [9]	LP403812, IC_50_ = 0.11 µM [51]	α-MT, IC_50_ = 250 µM [54]

## Data Availability

The original contributions presented in this study are included in the article/Supplementary Materials. Further inquiries can be directed to the corresponding author.

## Supplementary Materials

The following supporting information can be downloaded at: https://www.mdpi.com/article/10.3390/molecules30224412/s1. Refs. [25,49,51,52,53,55,60,61,62,63,64,65] are cited in the Supplementary Materials.
