# Supplementary material for: Unveiling the Structure of PROT and ATB0,+: Unique Members of the Glycine Transporter Subfamily
Source: Molecules. 2025 Nov 14;30(22):4412. doi: 10.3390/molecules30224412 (PMC12655857; doi:10.3390/molecules30224412)
Supplement: Supplementary file 1 [file molecules-30-04412-s001.zip › molecules-3945963-supplementary.pdf]

Supporting materials

Unveiling the Structure of PROT and ATB$^{0,+}$: Unique Members of the Glycine Transporter Subfamily

Dorota Stary [1,2,3] and Marek Bajda [1,*]

1 Department of Physicochemical Drug Analysis, Faculty of Pharmacy, Jagiellonian University Medical College, Medyczna 9, 30-688 Cracow, Poland; dorota.stary@doctoral.uj.edu.pl
2 Doctoral School of Medical and Health Sciences, Jagiellonian University Medical College, Św. Łazarza 16, 31-530 Cracow, Poland
3 Institute of Pharmacy and Food Chemistry, University of Würzburg, Am Hubland, 97074 Würzburg, Germany
* Correspondence: author: marek.bajda@uj.edu.pl

Table S1. Proteins from the PDB database selected as templates.

Template	State	PDB code	Organism	Method	Res. (Å)	Release date	Ref.
*a*LeuT	Outward-open	3FAF	*A. aeolicus*	X-ray diffraction	2.00	23.12.2008	[60]
		4MMB	*A. aeolicus VF5*	X-ray diffraction	2.25	16.10.2013	[61]
		4MM7	*A. aeolicus VF5*	X-ray diffraction	2.85	16.10.2013	
	Outward-occluded	2A65	*A. aeolicus VF5*	X-ray diffraction	1.65	02.08.2005	[49]
		2Q72	*A. aeolicus VF5*	X-ray diffraction	1.70	21.08.2007	
		2Q6H	*A. aeolicus VF5*	X-ray diffraction	1.85	21.08.2007	
*d*DAT	Outward-open	4XP4	*D. melan., M. musculus*	X-ray diffraction	2.80	06.05.2015	[62]
		4XP9	*D. melan., M. musculus*	X-ray diffraction	2.80	06.05.2015	
		6M2R	*D. melan., M. musculus*	X-ray diffraction	2.80	17.02.2021	[63]
	Outward-occluded	4XPH	*D. melan., M. musculus*	X-ray diffraction	2.90	06.05.2015	[62]
*h*SERT	Outward-open	5I6X	*H. sapiens., Mus mus.*	X-ray diffraction	3.14	13.04.2016	[64]
*h*GlyT1	Inward-open	6ZBV	*Homo sapiens*	X-ray diffraction	3.40	13.03.2021	[25]
*h*GAT1	Inward-occluded	7Y7W	*Homo sapiens*	Cryo-electron microscopy	2.40	26.04.2023	[65]
	Inward-open	7Y7Z	*Homo sapiens*	Cryo-electron microscopy	3.20	26.04.2023	[65]

A.aeolicus – Aquifex aeolicus, D. melan. – Drosophila melanogaster, M. musculus. – Mus musculus, H. sapiens – Homo sapiens. States were coloured: outward-open – blue, outward-occluded – green, inward-occluded – yellow, inward-open – orange.

Table S2. Assessment of PROT, ATB$^{0,+}$ models and selected templates with different tools.

PROT models						
Template PDB code	Tool	DopeScore	QMEAN	Ramachandran plots – in the core	Ramachandran – disallowed	Residues in disallowed region
4XP9	SWISSMODEL	-84978	-2.44	93.3	0.2	Ser457
4XP9	Modeller	-82242	-2.38	96.6	0.0	-
4XPH	SWISSMODEL	-84091	-2.87	95.7	0.4	Asn182, Arg420
2A65	Modeller	-78778	-4.57	93.1	0.2	Gln350
7Y7W	SWISSMODEL	-84413	-3.13	92.6	0.2	Phe460
7Y7W	Modeller	-81465	-2.64	96.1	0.2	Phe494
6ZBV	Modeller	-79027	-2.60	94.2	0.0	-

7Y7Z	SWISSMODEL	-82926	-3.52	94.1	0.0	-
ATB$^{0,+}$ models						
6M2R	Modeller	-82158	-3.69	94.2	0.2	Gln224
6M2R	SWISSMODEL	-87249	-4.43	90.9	0.4	Ser158, Gln187
4XPH	Modeller	-81797	-4.07	95.4	0.4	Gln224, Lys445
2A65	Modeller	-79428	-5.54	96.2	0.6	Leu113, Met443, Ala469
7Y7W	SWISSMODEL	-85938	-5.09	89.3	0.6	Lys90, His172, Trp192
7Y7W	Modeller	-83032	-3.58	94.2	0.0	-
6ZBV	Modeller	-81666	-4.22	92.4	0.6	Asp389, Trp484, Trp525
6ZBV	SWISSMODEL	-85022	-5.46	88.2	0.4	Ser158, Asp389,
Templates from PDB database (N- and C-terminus were omitted)						
4XP9	-	-85111	-2.32	94.4	0.0	-
6M2R	-	-84716	-2.82	92.4	0.0	-
4XPH	-	-84886	-2.85	94.9	0.0	-
2A65	-	-86822	-1.45	94.5	0.0	-
7Y7W	-	-85244	-2.89	92.6	0.0	-
6ZBV	-	-82540	-3.65	91.9	0.0	-
7Y7Z	-	-84038	-4.02	90.3	0.0	-

States were coloured: outward-open – blue, outward-occluded – green, inward-occluded – yellow, inward-open – orange.

Table S3. Structures of PROT inhibitors.

Ligand name	Structure	Ligand name	Structure	Ref.
**1**	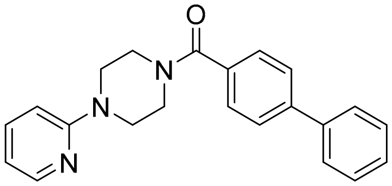	**30**	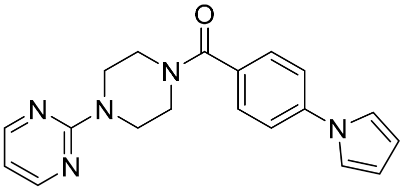	
**2**	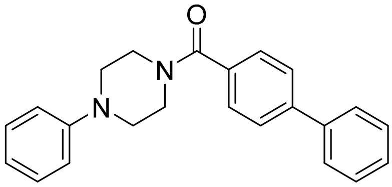	**31**	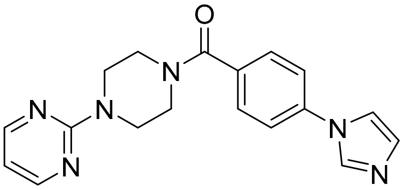	
**3**	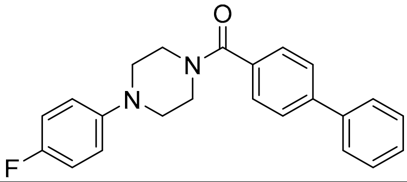	**32**	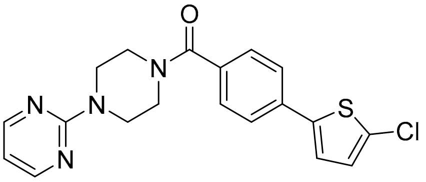	[52]
**4**	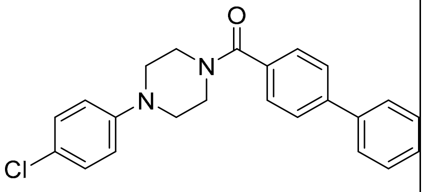	**33**	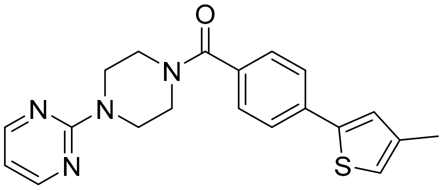	
**5**	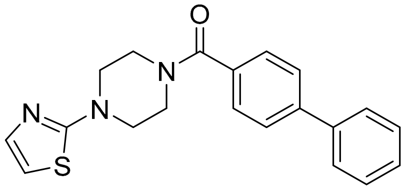	**34**	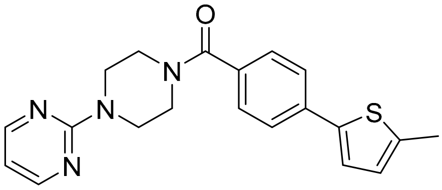	

**6**	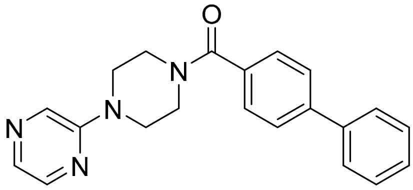	**37**	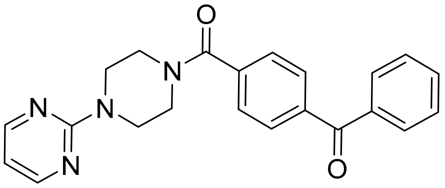	
**7**	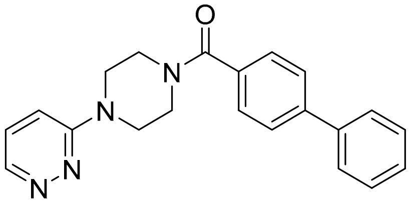	**38**	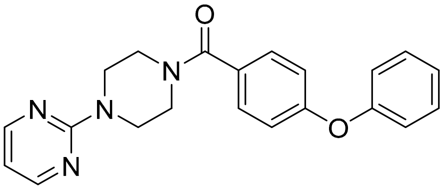	
**8**	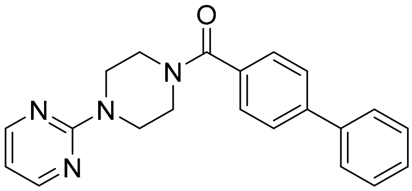	**39**	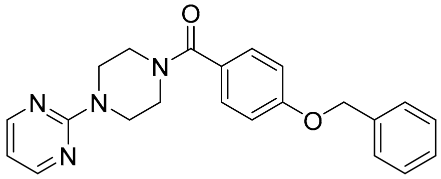	
**9**	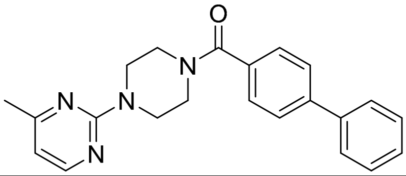	**40**	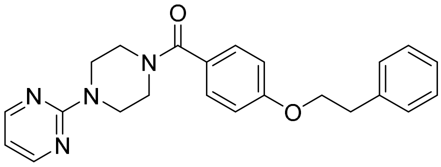	
**10**	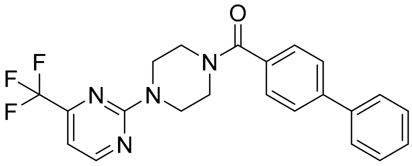	**41**	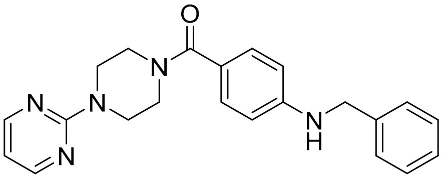	
**11**	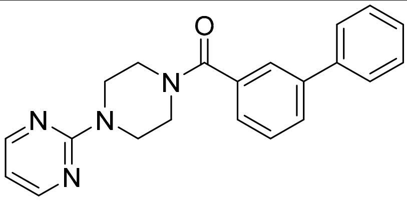	**42**	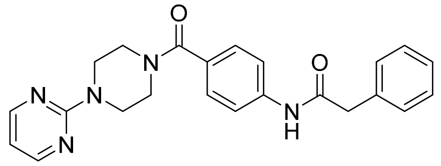	
**12**	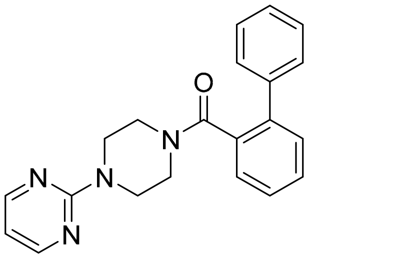	**43**	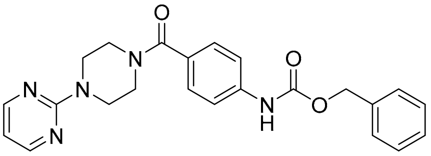	
**15**	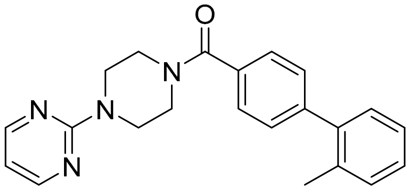	**44**	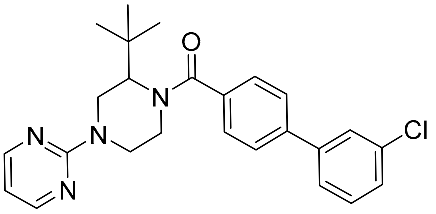	
**16**	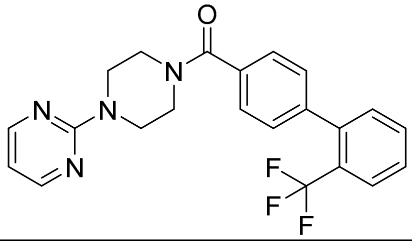	**45**	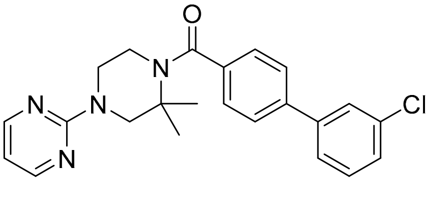	

**17**	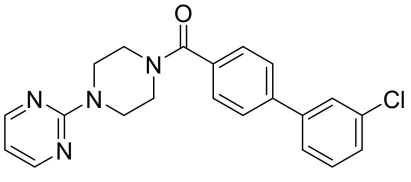	**46**	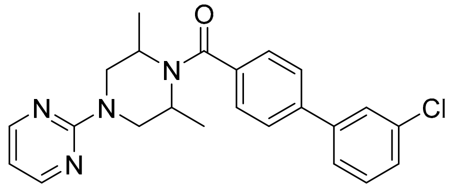	
**18**	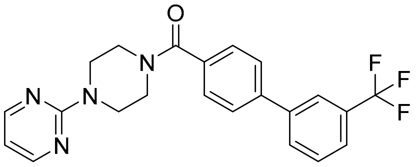	**47**	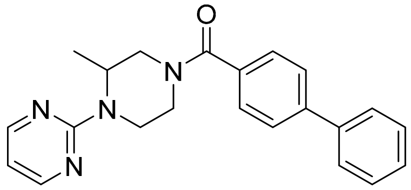	
**19**	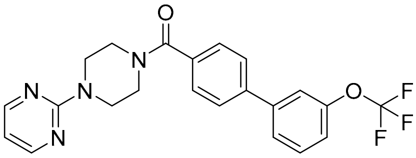	**48**	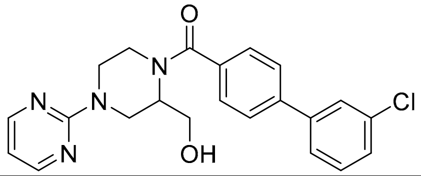	
**20**	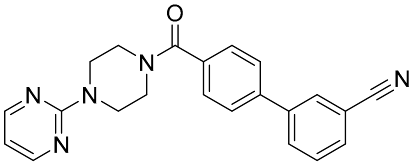	**49**	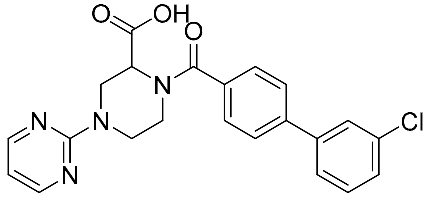	
**21**	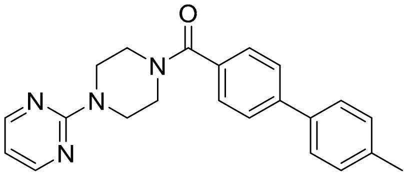	**50**	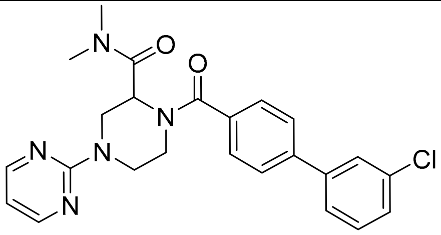	
**22**	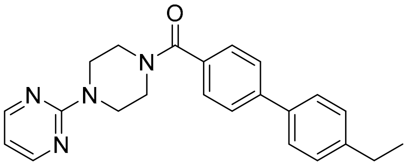	**51**	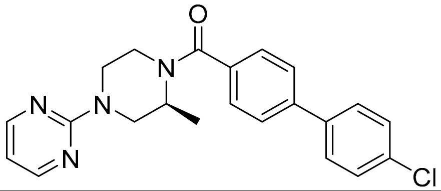	
**23**	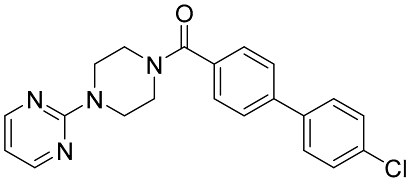	**52**	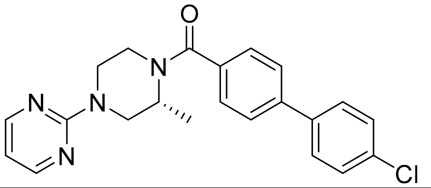	
**24**	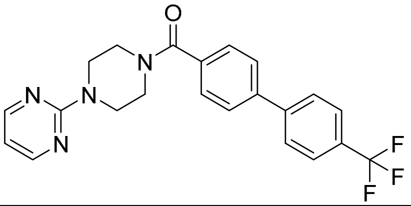	**53**	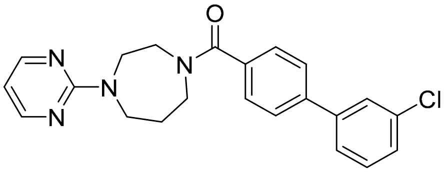	
**25**	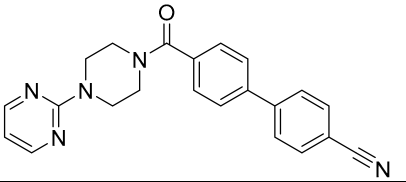	**54**	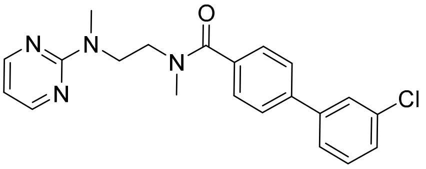	

**26**	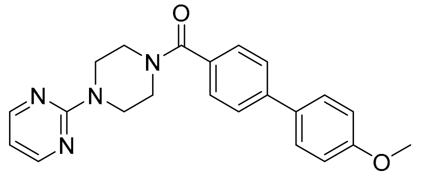	**55**	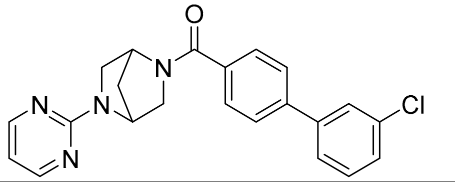	
**27**	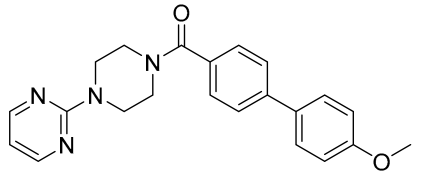	**56**	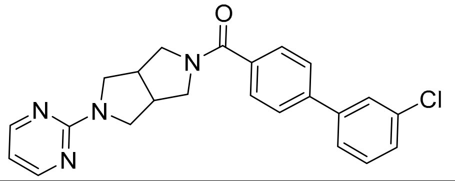	
**28**	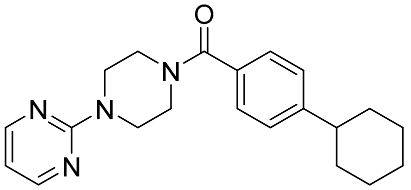	**57**	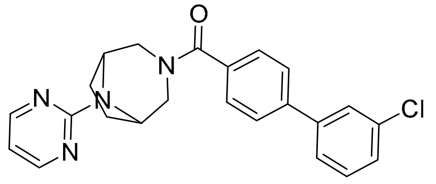	
**29**	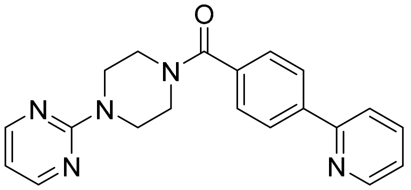	**58**	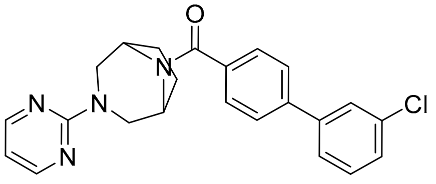	
**LP403812**	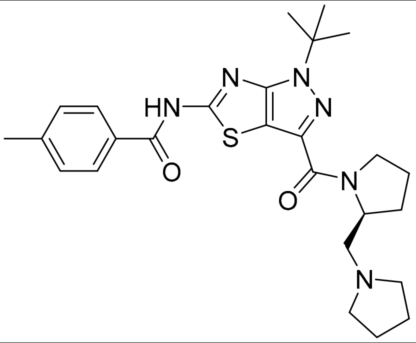	**LQFM215**	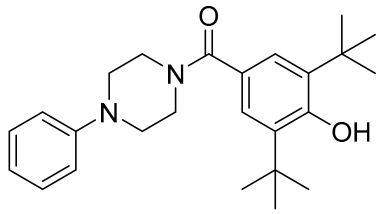	[51]
**LQFM216**	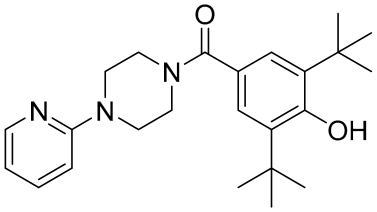	**LQFM217**	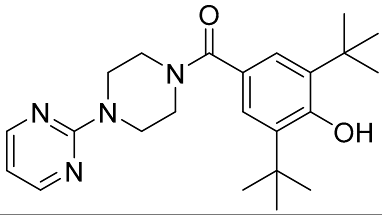	
**BP50**	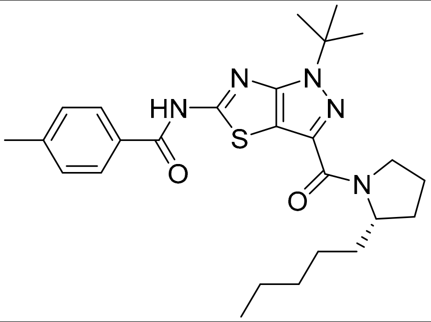			[53]
**Bitopertin**	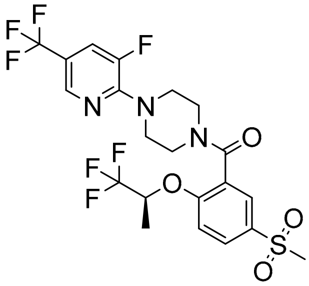			[55]

Table S4. Inhibitors docked to the PROT models in outward and inward open states, as well as within GlyT1 in inward-open state.

PROT					GlyT1
Ligand	IC_{50} [μM]	Ref.	Outward-open state	Inward-open state	Inward-open state
1	0.160	[52]	-4.87	-8.86	-6.68
2	1.480		-4.63	-7.54	-7.14
3	3.440		-4.07	-6.60	-6.87
4	10.000		-3.95	-8.87	-6.64
5	0.146		-4.88	-8.84	-8.19
6	1.300		-4.35	-7.26	-6.88
7	5.000		-4.77	-5.44	-
8	0.280		-4.93	-8.10	-6.73
9	0.270		-5.13	-5.15	-6.28
10	2.180		-4.08	-8.45	-6.07
11	1.880		-5.49	-8.83	-8.10
12	6.010		-4.91	-9.48	-7.66
15	0.653		-5.17	-8.16	-6.79
16	1.260		-5.29	-9.54	-7.50
17	0.036		-5.06	-8.18	-4.81
18	0.071		-6.66	-9.44	-6.42
19	0.073		-4.62	-7.27	-5.82
20	0.190		-5.05	-8.32	-6.58
21	0.034		-3.45	-5.61	-5.64
22	0.077		-	-5.84	-6.28
23	0.052		-4.15	-5.97	-6.29
24	0.076		-	-6.65	-
25	0.190		-	-6.08	-5.16
26	0.230		-5.44	-8.56	-5.90
27	0.260		-5.25	-6.92	-6.68
28	7.010		-5.39	-8.72	-6.37
29	7.080		-5.02	-8.23	-4.63
30	2.230		-5.40	-7.95	-6.78
31	10.000		-6.28	-7.84	-6.57
32	0.150		-5.04	-8.40	-6.33
33	0.034		-4.92	-8.28	-4.80
34	0.061		-3.79	-6.49	-3.92
37	2.700		-5.41	-8.48	-6.63
38	1.950		-4.90	-7.97	-6.26
39	0.270		-5.56	-7.28	-6.40
40	1.020		-3.65	-8.05	-5.60
41	0.870		-6.04	-7.98	-6.26
42	10.000		-3.39	-5.81	-5.34
43	10.000		-	-	-5.82
44	0.700		-4.34 (R)	-7.70 (R)	-5.07 (R)
			- (S)	-1.15 (S)	- (S)
45	0.053		-4.16	-2.93	-6.83

46	0.024		-4.57	-8.48	-6.73
47	0.477		-3.88 (R)	-	-
			-5.10 (S)	-5.28 (S)	-6.42 (S)
48	0.076		-4.89 (R)	-5.32 (R)	- (R)
			-5.38 (S)	- (S)	-6.76 (S)
49	0.128		-4.47 (R)	-7.12 (R)	-5.72 (R)
			-5.7 (S)	-8.58 (S)	-4.77 (S)
50	0.392		-5.13 (R)	-9.34 (R)	- (R)
			-3.48 (S)	-7.61 (S)	-5.56 (S)
51	0.021		-1.53	-6.93	-5.86
52	0.057		-5.20	-5.53	-
53	0.089		-4.59	-8.68	-7.05
54	0.586		-4.48	-8.35	-6.11
55	1.340		-5.26	-9.24	-6.43
56	0.036		-4.78	-8.64	-7.90
57	0.027		-5.71	-	-
58	0.018		-5.58	-9.43	-4.45
LP403812	0.110	[51]	-4.77	-7.01	-4.84
LQFM215	20.400		-4.99	-8.21	-6.82
LQFM216	31.500		-4.66	-8.16	-6.32
LQFM217	24.040		-4.68	-7.77	-6.86
BP50	-	[53]	-3.65	-6.18	-
Bitopertin	-	[55]	-5.82	-8.02	-6.97

Colour scale was adjusted in case of docking scores: blue (not beneficial) – white – red (the most beneficial). Docking to the occluded states within PROT were omitted due to lack of results.

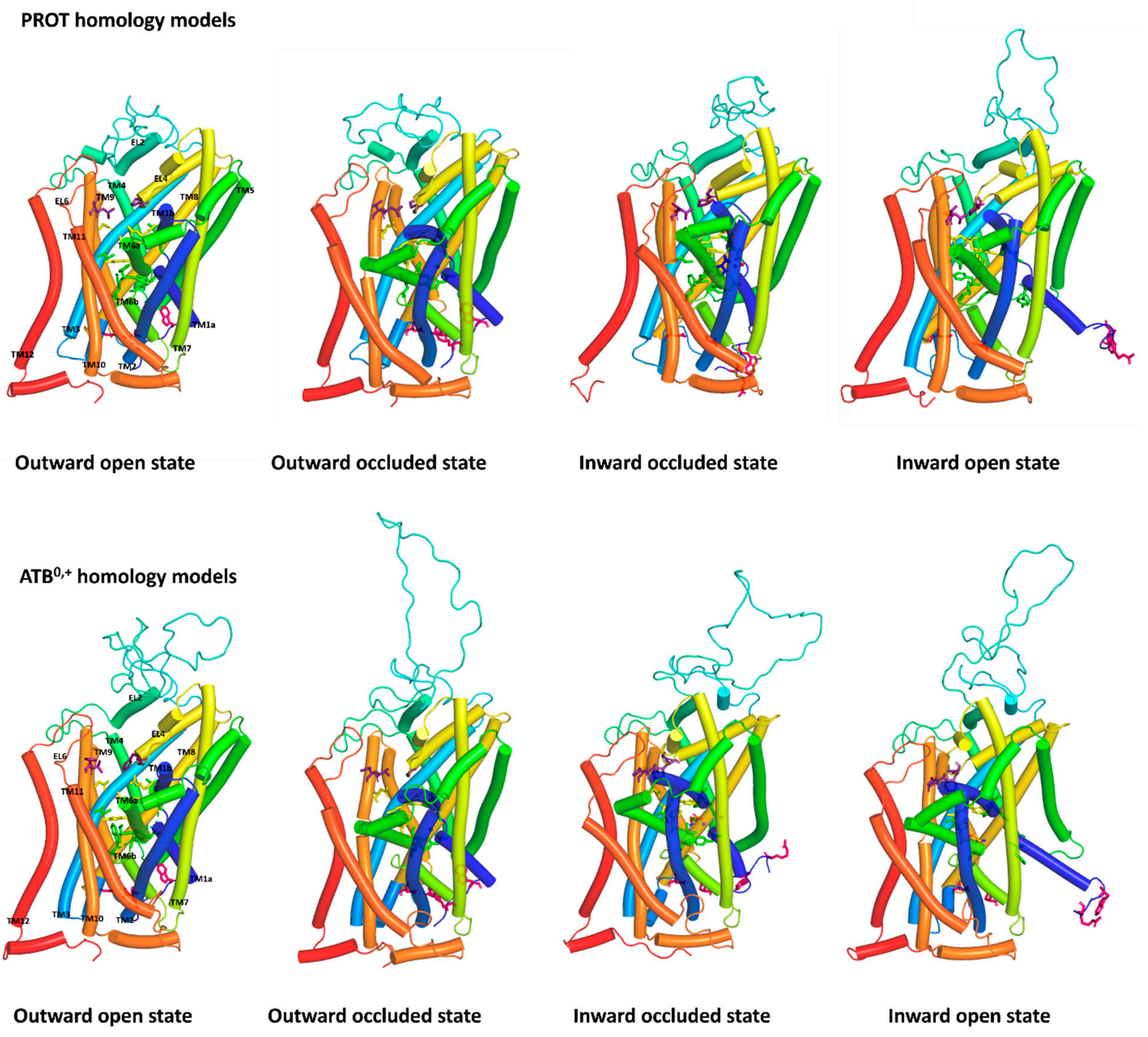

Figure S1. Models of PROT (upper panel) and ATB$^{0,+}$ (down panel) in different conformational states. Residues are marked with the colours: extracellular gate – yellow, intracellular gate – pink, S2 site binding site – violet, S1 binding site – green. Ions binding sites were omitted to clarity.

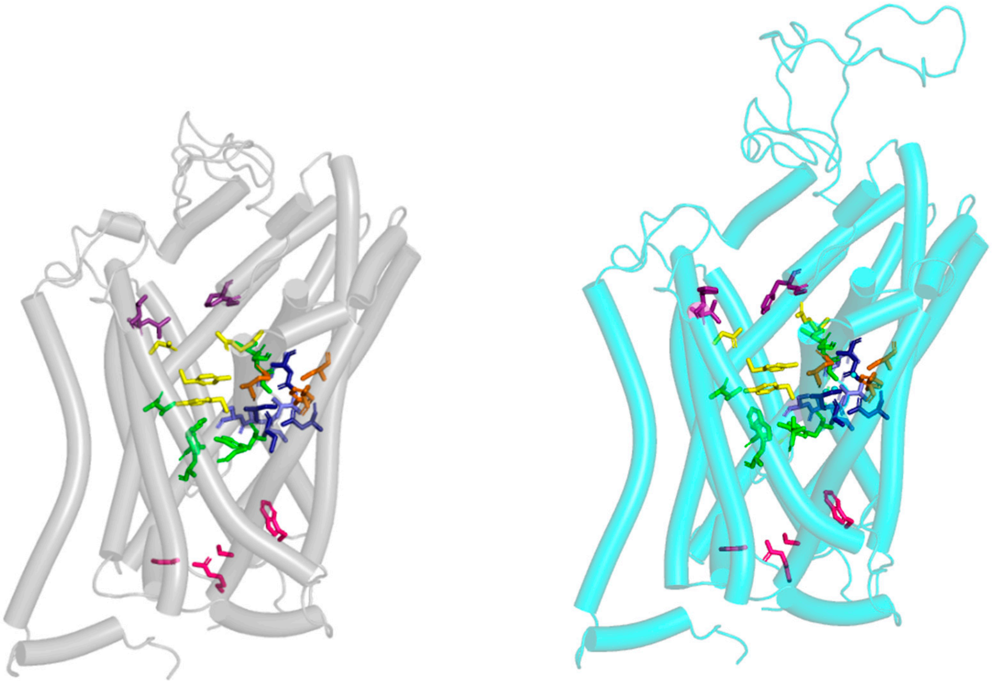

Figure S2. Overall structure of transporter models in outward open state: PROT model (template PDB code: 4XP9, tool: Modeller) – left panel, $ATB^{0,+}$ model (template PDB code: 6M2R) – right panel. Transmembrane domains of proteins are coloured as follows: PROT – grey, $ATB^{0,+}$ - light blue. Residues are marked with the colours: extracellular gate – yellow, intracellular gate – pink, Na^{+} 1 binding site – blue, Na^{+}2 binding site – light blue and Cl^{-} binding site – orange, S2 site binding site – violet, S1 binding site – green.

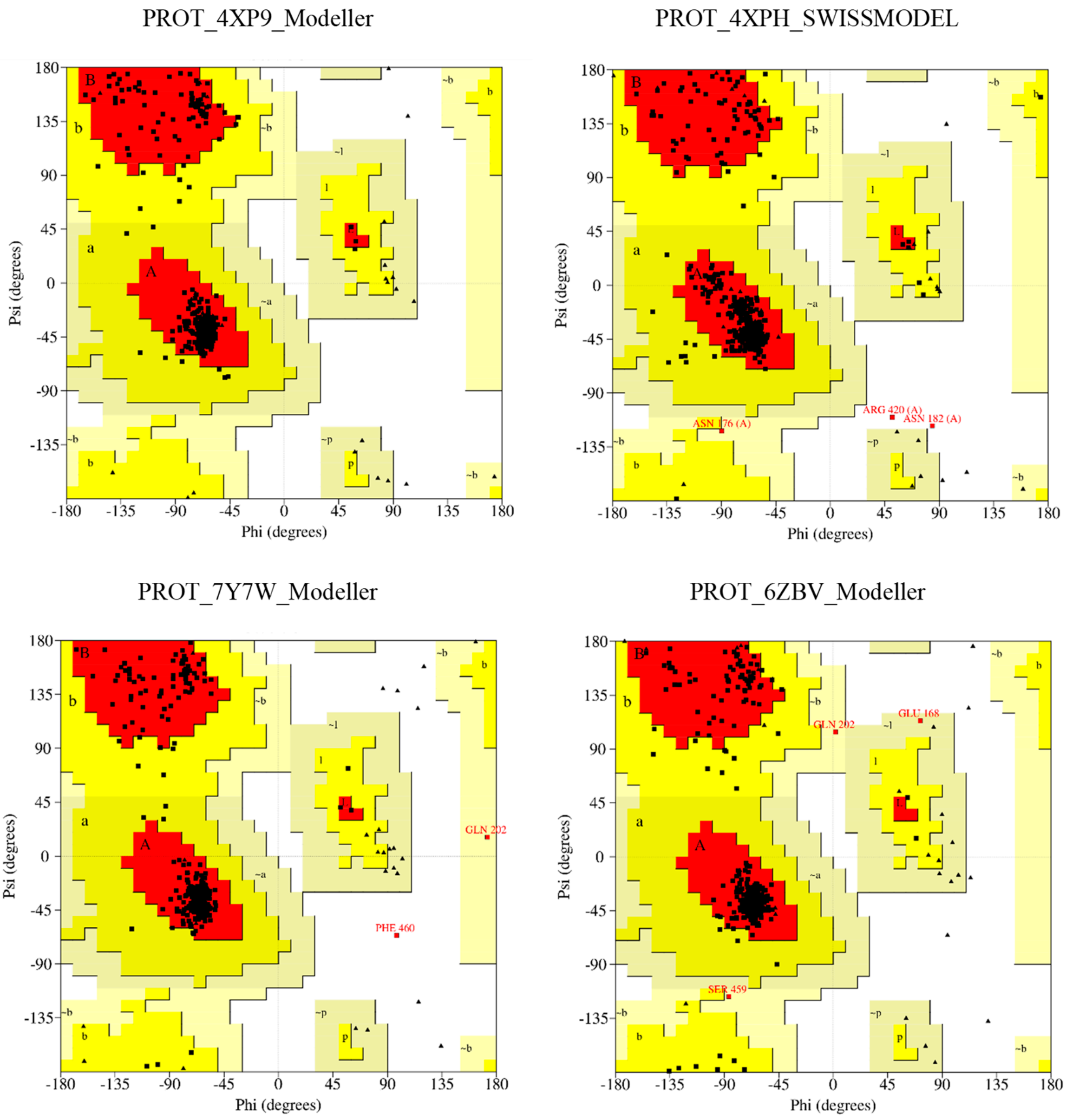

Figure S3. Assessment of PROT models with Ramachandran plots.

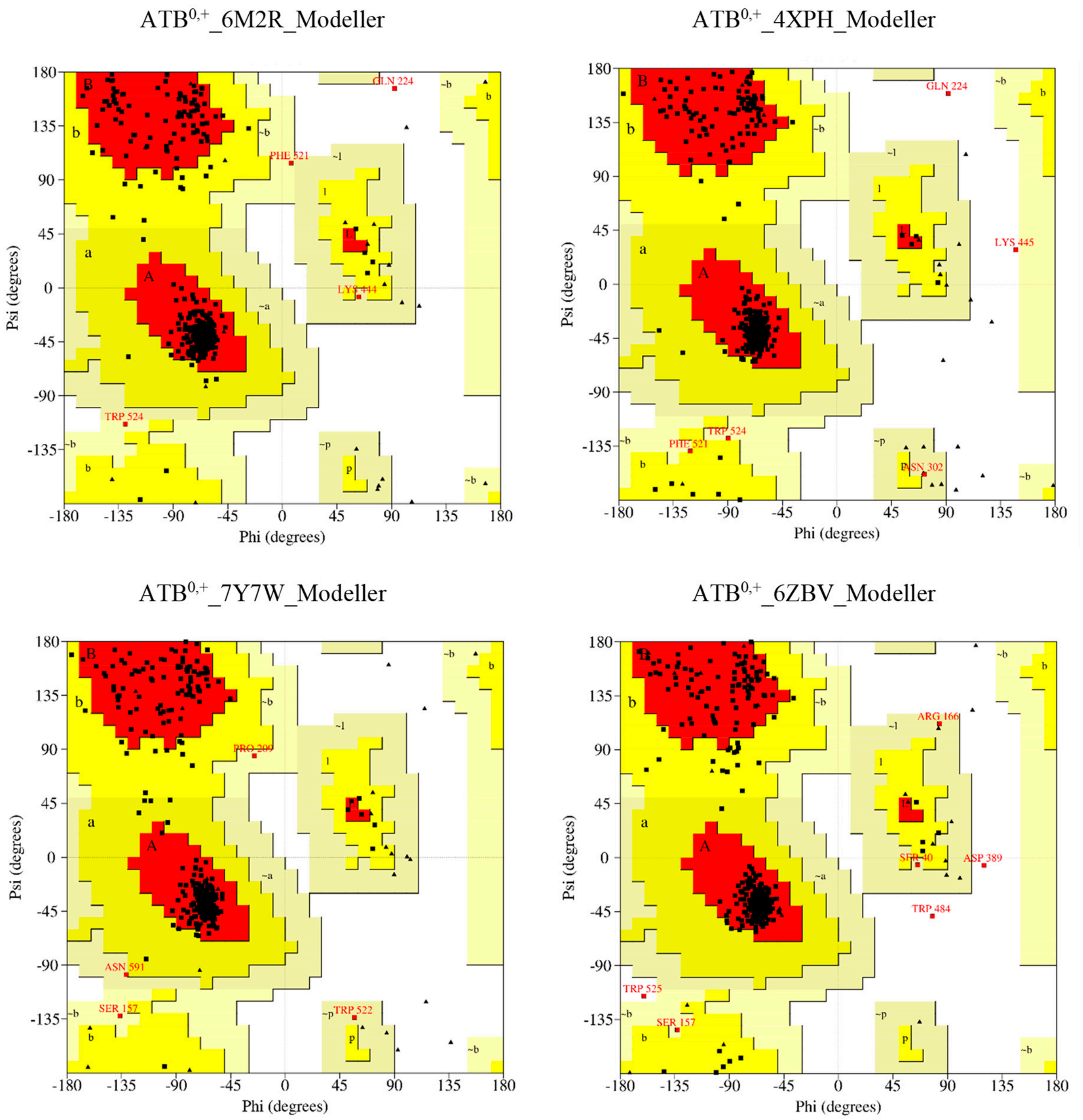

Figure S4. Assessment of ATB$^{0,+}$ models with Ramachandran plots.

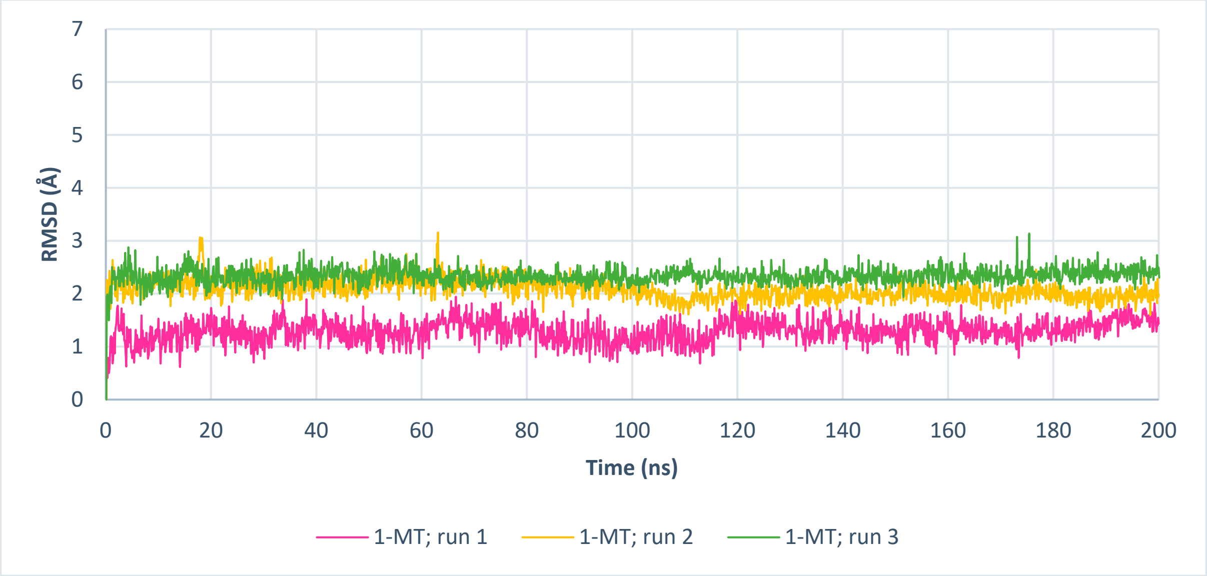

Figure S5. RMSD changes of 1-MT within $ATB^{0,+}$ model (template: 7Y7W, tool: Modeller) in inward-occluded state during molecular dynamics simulation.

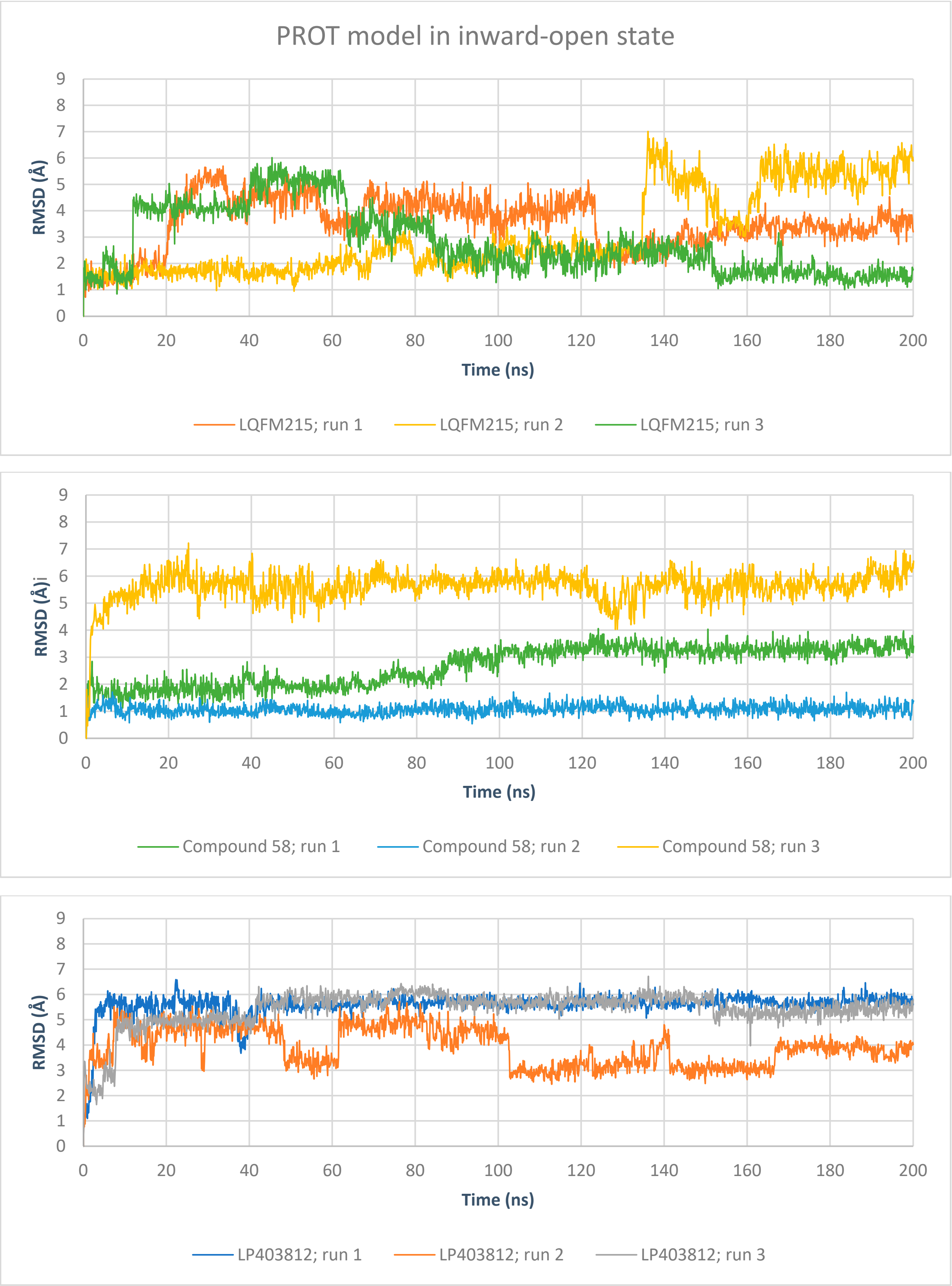

Figure S6. RMSD changes of inhibitors within PROT model (template: 6ZBV, tool: Modeller) in inward open state during molecular dynamics simulation: LQFM215 (upper panel), Compound 58 (middle panel), LP403812 (down panel).

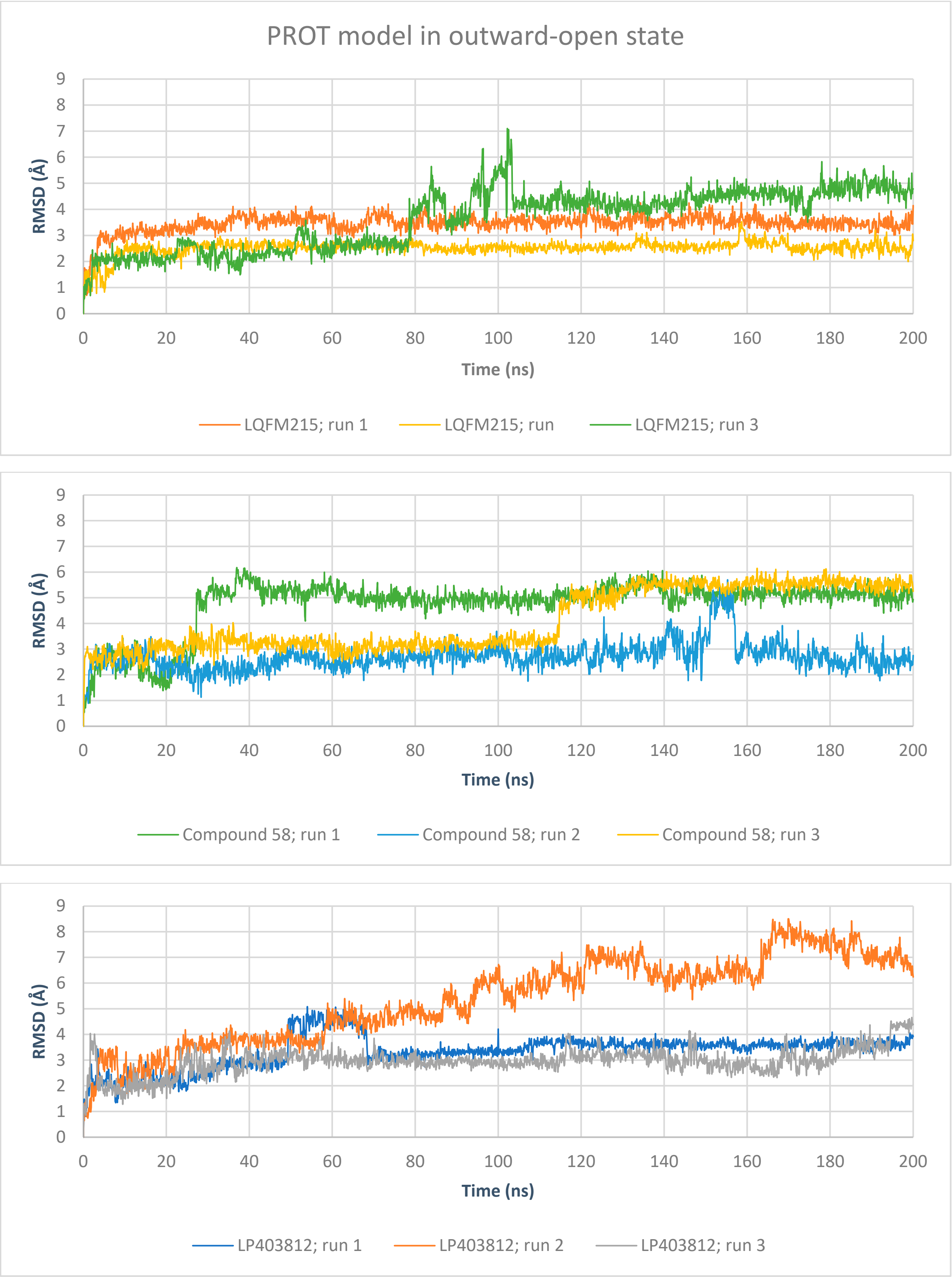

Figure S7. RMSD changes of inhibitors within PROT model (template: 4XP9, tool: Modeller) in outward open state during molecular dynamics simulation: LQFM215 (upper panel), Compound 58 (middle panel), LP403812 (down panel).

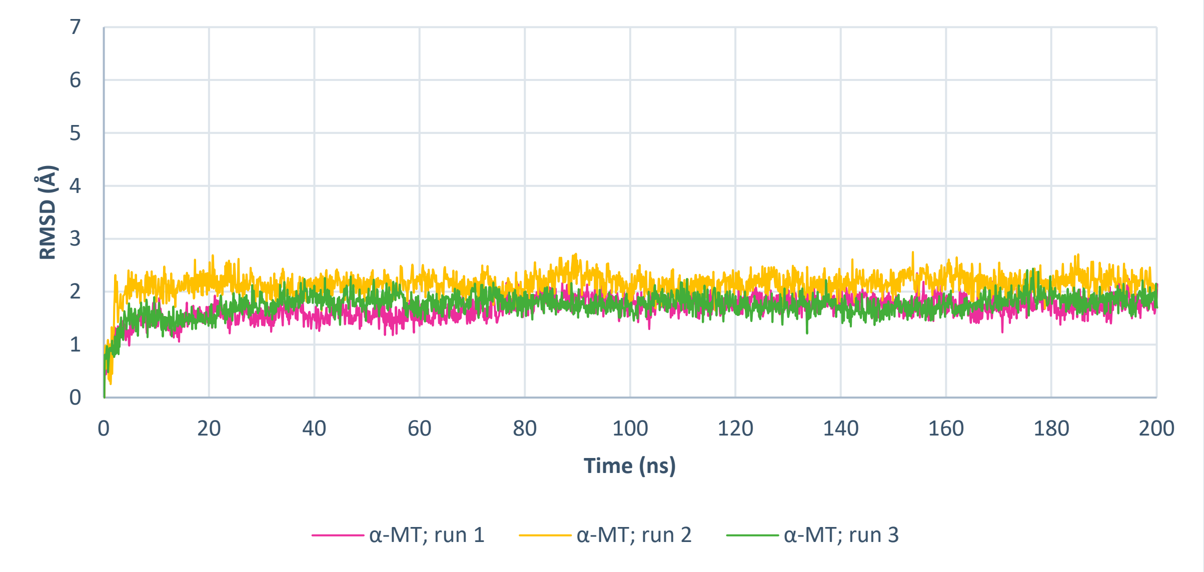

Figure S8. RMSD changes of α-MT within ATB$^{0,+}$ model (template: 7Y7W, tool: Modeller) in inward-occluded state during molecular dynamics simulation.

References

[25] A. Shahsavar, P. Stohler, G. Bourenkov, I. Zimmermann, M. Siegrist, W. Guba, E. Pinard, S. Sinning, M.A. Seeger, T.R. Schneider, R.J.P. Dawson, P. Nissen, Structural insights into the inhibition of glycine reuptake, Nature 2021 591:7851 591 (2021) 677–681. https://doi.org/10.1038/s41586-021-03274-z.

[49] A. Yamashita, S.K. Singh, T. Kawate, Y. Jin, E. Gouaux, Crystal structure of a bacterial homologue of Na+/Cl --dependent neurotransmitter transporters, Nature 437 (2005) 215–223. https://doi.org/10.1038/nature03978.

[51] X.C. Yu, W. Zhang, A. Oldham, E. Buxton, S. Patel, N. Nghi, D. Tran, T.H. Lanthorn, C. Bomont, Z.C. Shi, Q. Liu, Discovery and characterization of potent small molecule inhibitors of the high affinity proline transporter, Neurosci Lett 451 (2009) 212–216. https://doi.org/10.1016/j.neulet.2009.01.018.

[52] G.G. Zipp, J. Barbosa, M.A. Green, K.M. Terranova, C. Fink, X.C. Yu, A. Nouraldeen, A. Wilson, K. Savelieva, T.H. Lanthorn, S. David Kimball, Novel inhibitors of the high-affinity l-proline transporter as potential therapeutic agents for the treatment of cognitive disorders, Bioorg Med Chem Lett 24 (2014) 3886–3890. https://doi.org/10.1016/J.BMCL.2014.06.049.

[53] M.-M. Li, H. Huang, Y. Pu, W. Tian, Y. Deng, J. Lu, A close look into the biological and synthetic aspects of fused pyrazole derivatives, Eur J Med Chem 243 (2022) 114739. https://doi.org/10.1016/j.ejmech.2022.114739.

[55] G. Almeida De Carvalho, P.M. Tambwe, L. Rodrigues, C. Nascimento, B. Kelly, P. Campos, R.A. Chiareli, G. Pereira, N. Junior, R. Menegatti, R. Santiago Gomez, M. Cunha, X. Pinto, In silico evidence of bitopertin's broad interactions within the SLC6 transporter family, Journal of Pharmacy and Pharmacology (2024) 1–13. https://doi.org/10.1093/JPP/RGAE051.

[60] S.K. Singh, C.L. Piscitelli, A. Yamashita, E. Gouaux, A competitive inhibitor traps LeuT in an open-to-out conformation, Science (1979) 322 (2008) 1655–1661. https://doi.org/10.1126/science.1166777.

[61] H. Wang, A. Goehring, K.H. Wang, A. Penmatsa, R. Ressler, E. Gouaux, Structural basis for action by diverse antidepressants on biogenic amine transporters, Nature 503 (2013) 141–145. https://doi.org/10.1038/nature12648.

[62] K.H. Wang, A. Penmatsa, E. Gouaux, Neurotransmitter and psychostimulant recognition by the dopamine transporter, Nature 521 (2015) 322–327. https://doi.org/10.1038/nature14431.

[63] S. Pidathala, A.K. Mallela, D. Joseph, A. Penmatsa, Structural basis of norepinephrine recognition and transport inhibition in neurotransmitter transporters, Nat Commun 12 (2021). https://doi.org/10.1038/s41467-021-22385-9.

[64] J.A. Coleman, E.M. Green, E. Gouaux, X-ray structures and mechanism of the human serotonin transporter, Nature 532 (2016) 334–339. https://doi.org/10.1038/nature17629.

[65] A. Zhu, J. Huang, F. Kong, J. Tan, J. Lei, Y. Yuan, C. Yan, Molecular basis for substrate recognition and transport of human GABA transporter GAT1, Nat Struct Mol Biol 30 (2023). https://doi.org/10.1038/s41594-023-00983-z.
